# Tau is a receptor with low affinity for glucocorticoids and is required for glucocorticoid-induced bone loss

**DOI:** 10.1038/s41422-024-01016-0

**Published:** 2025-01-02

**Authors:** Wenyu Fu, Meng Chen, Kaidi Wang, Yujianan Chen, Yazhou Cui, Yangli Xie, Zi-Ning Lei, Wenhuo Hu, Guodong Sun, Guiwu Huang, Chaopeng He, Jackie Fretz, Aubryanna Hettinghouse, Ronghan Liu, Xianyi Cai, Mingshuang Zhang, Yuehong Chen, Nan Jiang, Minchun He, Daniel H. Wiznia, Huiyun Xu, Zhe-Sheng Chen, Lin Chen, Kanglai Tang, Hong Zhou, Chuan-Ju Liu

**Affiliations:** 1 Department of Orthopaedics and Rehabilitation, Yale University School of Medicine, New Haven, CT USA; 2 Department of Orthopaedic Surgery, New York University Grossman School of Medicine, New York, NY USA; 3 Department of Orthopedics/Sports Medicine Center, Southwest Hospital, Third Military Medical University, Chongqing, China; 4 Biomedical Sciences College & Shandong Medicinal Biotechnology Centre, Shandong First Medical University & Shandong Academy of Medical Sciences, Jinan, Shandong China; 5 Laboratory of Wound Repair and Rehabilitation Medicine, State Key Laboratory of Trauma, Burns and Combined Injury, Daping Hospital, Army Medical University, Chongqing, China; 6 Department of Pharmaceutical Science, College of Pharmacy and Health Sciences, St. John’s University, New York, NY USA; 7 Human Oncology and Pathogenesis Program, Memorial Sloan Kettering Cancer Center; Marie-Josée and Henry R. Kravis Center for Molecular Oncology, Memorial Sloan Kettering Cancer Center, New York, NY USA; 8 School of Life Sciences, Northwestern Polytechnical University, Xi’an, Shaanxi China; 9 Bone Research Program, ANZAC Research Institute, The University of Sydney, Sydney, NSW Australia; 10 Department of Cell Biology, New York University Grossman School of Medicine, New York, NY USA

**Keywords:** Mechanisms of disease, Stress signalling

## Abstract

Glucocorticoids (GCs) are the most prescribed anti-inflammatory and immunosuppressive drugs. However, their use is often limited by substantial side effects, such as GC-induced osteoporosis (GIO) with the underlying mechanisms still not fully understood. In this study, we identify Tau as a low-affinity binding receptor for GCs that plays a crucial role in GIO. Tau deficiency largely abolished bone loss induced by high-dose dexamethasone, a synthetic GC, in both inflammatory arthritis and GIO models. Furthermore, TRx0237, a Tau inhibitor identified from an FDA-approved drug library, effectively prevented GIO. Notably, combinatorial administration of TRx0237 and dexamethasone completely overcame the osteoporosis adverse effect of dexamethasone in treating inflammatory arthritis. These findings present Tau as a previously unrecognized GC receptor with low affinity, and provide potential strategies to mitigate a spectrum of GC-related adverse effects, particularly osteoporosis.

## Introduction

The discovery of glucocorticoids (GCs) therapeutic effects against rheumatoid arthritis (RA) by Hench and colleagues led to the Nobel Prize in Physiology and Medicine in 1950^1^ and has revolutionized clinical practice for treating various inflammatory, autoimmune, and neoplastic diseases owing to GC’s potent anti-inflammatory and immunosuppressive actions.^2,3^ Since then, synthetic GCs, such as dexamethasone and prednisolone, represent the most widely prescribed drugs in the world.^4,5^ Recently, high-dose dexamethasone was a crucial treatment to people with advanced COVID-19.^6^ Although attractive, pharmacologic GCs treatment, particularly with high dosage and prolonged usage, can cause a wide range of side effects, including iatrogenic insulin resistance, high blood glucose, disorders of lipid metabolism, myopathy, increased risk of infection, and GC-induced osteoporosis (GIO), the most common cause of secondary osteoporosis.^3,7^ GIO is a consequence of increased bone resorption and decreased bone formation, the underlying mechanisms are intricate and regulated by a complex network of both local and systemic factors.^8–11^

The concomitance of therapeutic and deleterious impacts of GCs drives the research focus in two directions: one is to unravel the mechanisms underlying GCs therapeutic anti-inflammatory and immunosuppressive actions, and the second is to search for the knowledge behind the side effects of prolonged exposure to pharmacological concentrations of GCs, including GIO. It is well known that GCs bind to the canonical GC receptor (GR)^12^ with high affinity, which is at the apex of the regulatory network responsible for GCs’ anti-inflammatory and immunosuppressive actions.^2,4,13^ Selective GC actions or ligand-selective GR activators to maintain its anti-inflammatory while limit its skeletal action was the therapeutic goal for the past over 60 years.^14,15^ Unfortunately, there is no breakthrough up to date. Therefore, there is an urgent, unmet need to explore the mechanisms behind adverse effects associated with the high dosage and prolonged usage of GCs. From a different perspective, we report herein that therapeutic effects and side effects of GCs are mediated by separate mechanisms through binding to distinct receptors with different affinities. Using various approaches for isolating and characterizing the binding receptors of GCs, as well as employing genetically modified cell and animal models, we discovered Tau as a low-affinity receptor for GCs, required for GC-stimulated osteoclastogenesis and osteoporosis. Subsequent assays identified TTBK1 kinase and transcription factor NF-κB p50 as two key downstream mediators of GCs/Tau signaling in GC-dependent osteoclastogenesis and osteoporosis.

## Results

### Tau is a low-affinity receptor of GCs

**Fig. 1:**
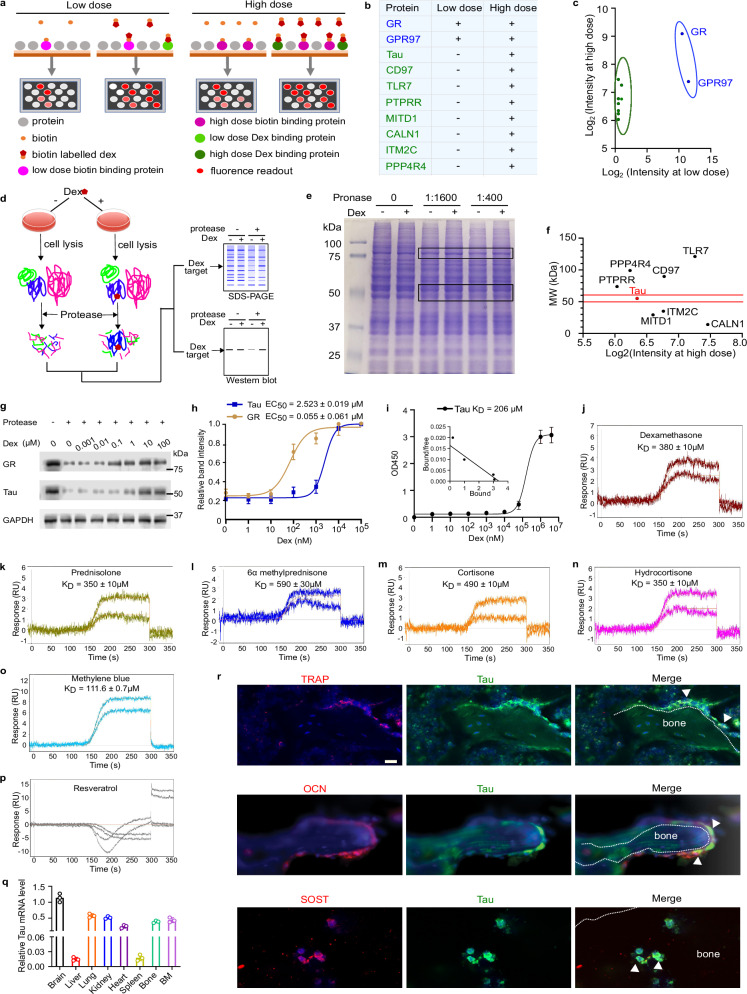
High-dose dexamethasone binds to Tau. **a** The schematic diagram of human proteome microarray, which contains over 20,000 individual proteins printed in duplicate, to identify binding partners of high-dose and low-dose dexamethasone, respectively. **b**, **c** Human proteome microarray analysis reveals the proteins (in blue) which bind to both low and high-dose dexamethasone, and proteins (in green) which selectively bind to high-dose dexamethasone. **d** Principle of DARTS assay for the isolation of proteins protected from degradation by dexamethasone. **e** Dexamethasone protects two groups of protein bands (highlighted in black rectangle) from degradation in DARTS using whole cell lysate from dexamethasone-treated Raw264.7 cells coupled with Coomassie blue staining. **f** Molecular weight (MW) plot of putative high-dose dexamethasone-binding proteins identified by human proteome microarray analysis. **g** The protective effects of serial doses of dexamethasone on Tau and GR from digestion by protease are evaluated by DARTS coupled with immunoblotting. GAPDH is resistant to protease under the condition and serves as a loading indicator. Representative image is shown (*n* = 3). **h** Quantification of Tau and GR stability treated with serial dosages of dexamethasone assayed by DARTS (*n* = 3). **i** The interaction between dexamethasone and Tau, assayed by solid phase binding. 10 mM Tau was coated to the plate, and a serial dilution of biotin-labeled dexamethasone was added, followed by incubation with HRP-labeled Streptavidin and its substrate (*n* = 3). Inset shows the Scatchard plot analysis for *K*_D_ value calculation. **j**–**p** One-step kinetic SPR assay for binding of Tau to different GCs, as indicated. **q** qRT-PCR analysis of *Tau* mRNA levels in different tissues, as indicated (*n* = 3). **r** Double-immunoflurorescence staining of femur section using antibodies against Tau (green) and TRAP, osteocalcin (OCN) and sclerostin (SOST) (red). DAPI stains nuclei. Arrows indicate positive staining cells. Scale bar = 20 µm. BM, bone marrow. Data are means ± SD in **h**, **i**, **q**.

To identify potential and previously unrecognized receptor(s) of GCs, with a special focus on the low-affinity receptor(s) that are distinct from the known high-affinity receptor GR, we probed genome-wide human protein arrays which contain over 20,000 individually printed proteins that are coded by more than 16,000 human genes, with low and high doses of biotin-labeled dexamethasone (Fig. 1a).^16–18^ The results revealed that dexamethasone bound to GR and GPR97, two known receptors of dexamethasone,^12,19^ with high affinity given that both low- and high-dose dexamethasone could bind to them (Fig. 1b, c). However, mineralocorticoid receptor, another high-affinity receptor of GCs, did not show up as a potential binder in our current protein array screening. Considering that the interactions between dexamethasone and its low-affinity receptor(s) occur only with high-dose dexamethasone, we compared the binding profiles between low and high-dose dexamethasone focusing on the proteins which selectively bound to high-dose dexamethasone, which led to the identification of 8 proteins (Fig. 1b, c).

We next sought to identify the binding partners of high-dose dexamethasone under physiological conditions through a drug affinity responsive target stability (DARTS)^20^ approach implementing Raw264.7 macrophages (Fig. 1d). After incubation with high-dose dexamethasone, the cells were lysed and subjected to protease digestion, followed by the detection of the target bands via Coomassie blue staining. As shown in Fig. 1e, two groups of protein bands were protected: group 1 bands were ~90 kDa, which was close to the molecular weight of GR; whereas another group of protected bands was 50 kDa or so. These results, together with the isolation of 8 high-dose dexamethasone-binding partners with the aforementioned proteome screening, led to the identification of Tau, a group of isoforms produced by alternative splicing with molecular weight of ~50 kDa,^21,22^ as the only candidate that may bind to high-dose dexamethasone in both protein chips and live cells (Fig. 1f).

We further confirmed and compared the interactions of dexamethasone/Tau and dexamethasone/GR by treating Raw264.7 macrophages with serial concentrations of dexamethasone followed by a DARTS assay coupled with immunoblotting with antibodies specifically against GR and Tau, respectively. The DARTS assay demonstrated that 10 µM high-dose dexamethasone was needed to obtain the clear protection of Tau from protease-mediated degradation, whereas protection of GR degradation could be seen from a dose as low as 10 nM dexamethasone (Fig. 1g, h). The estimated dissociation constant (*K*_D_) values of dexamethasone/Tau and dexamethasone/GR were 2.523 µM and 0.055 µM, respectively. Biophysical methods, including solid phase binding and one-step kinetic surface plasmon resonance (SPR) assay, further gave estimated *K*_D_ values of 206 µM and 380 µM for the interaction between dexamethasone and Tau, respectively (Fig. 1i, j). To be noted, the SPR assay indicated that both endogenous GCs, cortisone and hydrocortisone, and synthetic GCs, including prednisolone and methylprednisone, displayed comparable binding affinity to dexamethasone with Tau (Fig. 1j–n).^23^ Resveratrol, a chemical with a structure unrelated to that of GCs, was used as a negative control and did not show any binding to Tau, whereas methylene blue (MB), a chemical known to bind Tau,^24^ was chosen as a positive control and clearly bound to Tau in our SPR assay (Fig. 1o, p). Of note, DARTS assay with live cells, solid phase binding and SPR assay with purified Tau protein gave different estimated binding affinity with dexamethasone, suggesting possible involvements of co-factors in promoting the binding of dexamethasone to Tau in the cells.

We next sought to identify the domain of Tau responsible for binding to dexamethasone by generating serial deletion constructs of Tau fused to FLAG tag (Supplementary information, Fig. S1a, b). DARTS assay with these constructs demonstrated that dexamethasone failed to protect the Tau deletion mutants lacking the proline-rich region from protease-mediated degradation (Supplementary information, Fig. S1c), indicating that the proline-rich region of Tau was required for its association with high-dose dexamethasone.^25^ Subsequent molecular docking simulations revealed that dexamethasone and prednisolone had similar binding affinities and binding positions with Tau, exhibiting hydrophilic interactions with the amino acid residues 190–236 located within the proline-rich region of Tau (Supplementary information, Fig. S1d–h). In addition, both GCs formed identical hydrogen bonding interactions with Asp193, Thr212, Lys225, and Ser238 on Tau. Correspondingly, DARTS assay with *Tau* knockout Raw264.7 cells transfected with Tau point mutants of these residues revealed that Thr212 on Tau was the most critical amino acid for interaction with high-dose dexamethasone (Supplementary information, Fig. S1i, j). Taken together, these results demonstrate that Tau binds to high-dose GCs as a previously unrecognized receptor with low affinity.

### Tau deficiency abolishes dexamethasone-induced bone resorption in inflammatory arthritis

Tau is well known as a brain protein that promotes microtubule assembly. We found that Tau was widely expressed in many tissues in 3-month-old mice besides the brain, at relatively high levels in the heart, lung, kidney, bone and bone marrow, and at low levels in the liver and spleen (Fig. 1q). In addition, double-immunofluorescence staining of bone sections from 3-month-old mice revealed that Tau was expressed in Tartrate-resistant acid phosphatase (TRAP)^+^ osteoclasts, osteocalcin^+^ osteoblasts, and sclerostin^+^ osteocytes (Fig. 1r). qRT-PCR also demonstrated that Tau was expressed in the osteoclasts, osteoblasts and osteocytes obtained from wild-type (WT) mice (Supplementary information, Fig. S2a).

**Fig. 2:**
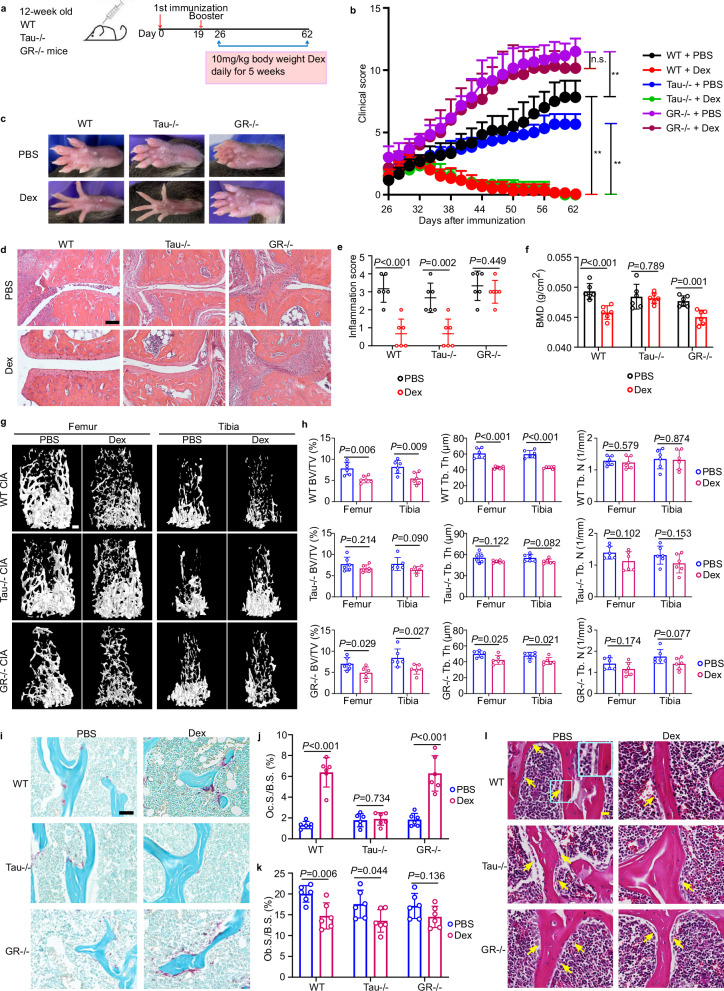
Tau deficiency abolishes high-dose dexamethasone-induced osteoporosis in CIA. **a** Scheme of experimental strategy to analyze the effects of Tau deficiency on dexamethasone-induced bone loss in inflammatory arthritis model. **b**, **c** Clinical arthritis scores (**b**) and images of paw (**c**) in WT, *Tau*^*−/−*^ and *GR*^*−/−*^ male mice with CIA treated with or without dexamethasone for 5 weeks (*n* = 6 mice for each group). **d**, **e** Representative images of hematoxylin and eosin (H&E) staining (**d**), and quantification of histomorphometric analysis of synovial inflammation (**e**) of ankle joints from the indicated mice (*n* = 6 mice for each group). **f** Whole body bone mineral density in WT, *Tau*^*−/−*^ and *GR*^*−/−*^ male mice with CIA treated with or without dexamethasone for 5 weeks, measured by DEXA scanning (*n* = 8 mice for each group). **g**, **h** Representative reconstructed 3D micro-CT images (**g**), and quantification of BV/TV, Tb.Th and Tb.N (**h**) of femur and tibia trabecular bone of WT, *Tau*^*−/−*^ and *GR*^*−/−*^ male CIA mice with the indicated treatment (*n* = 6 mice for each group). Scale bar = 250 µm in **g**. **i**, **j** Representative TRAP staining image (**i**), and quantification of TRAP^+^ osteoclast surface per bone surface (Oc.S./B.S.) (**j**) of femur distal metaphysis of WT, *Tau*^*−/−*^ and *GR*^*−/−*^ male mice with CIA in the same experiment (*n* = 6 mice for each group). Scale bar = 50 µm in **i**. **k**. Quantification of osteoblast surface per bone surface (Ob.S./B.S.) in the indicated mice. **l** Representative H&E staining images of femur showing osteoblasts (yellow arrows) on the trabecular bone. The area defined by blue rectangle on the representative image is enlarged as inset. Scale bar = 20 µm. Data are means ± SD. *P* values are calculated by two-tailed unpaired Student’s *t*-test (**b**, **e**, **f**, **h**, **j**, **k**).

Long-term pharmacological use of GCs causes osteoporosis, the most common side effect of GCs therapy in RA.^26^ Therefore, we first asked a question whether dexamethasone/Tau interaction was involved in dexamethasone-mediated osteoporosis with the collagen-induced arthritis (CIA) model, the widely-used, clinically-relevant inflammatory and autoimmune arthritis model (Fig. 2a).^27^

We generated inducible global *GR* knockout (*GR*^*−/−*^) mice by breeding *GR*^*flox/flox*^ mice^28^ with *Rosa26a-Cre*^*ERT2*^ mice in which Cre-mediated recombination was induced by tamoxifen.^29^ Due to the unavailability of *Tau*^*flox/flox*^ for generating conditional *Tau*^*−/−*^ mice, constitutive *Tau*^*−/−*^^30^ mice were employed in the current study. The deletion of Tau and GR in bone cell types, including osteoclasts, obteoblasts and osteocytes, in *Tau*^*−/−*^ and *GR*^*−/−*^ mice was confirmed by qRT-PCR (Supplementary information, Fig. S2a). GR deficiency rendered mice highly susceptible to CIA and impaired dexamethasone’s anti-inflammation action (Fig. 2b–e), which is in line with previous reports.^31,32^ In contrast, Tau deficiency resulted in reduced inflammation in CIA mice and did not impair dexamethasone-mediated suppression of inflammation (Fig. 2b–e). Strikingly, dexamethasone induced significant bone loss in WT and *GR*^*−/−*^ CIA mice, but not in *Tau*^*−/−*^ CIA mice (Fig. 2f). Microcomputed tomography (µCT) analysis and TRAP staining revealed that dexamethasone led to significant femoral and tibia trabecular bone loss by promoting osteoclast activity in both WT and *GR*^*−/−*^ CIA mice. However, the observed dexamethasone-induced bone loss was to a lesser extent in *GR*^*−/−*^ CIA mice than in WT CIA mice. Tau deficiency abolished dexamethasone-induced osteoclast activity and the resultant bone loss (Fig. 2g–j). In contrast, osteoblast surface values were significantly decreased in both of WT and *Tau*^*−/−*^ CIA mice treated with dexamethasone, but not in dexamethasone-treated *GR*^*−/−*^ CIA mice, indicating dexamethasone reduced osteoblast surface mainly through GR (Fig. 2k, l; Supplementary information, Fig. S2d, e). Nevertheless, overall µCT analysis revealed that high-dose dexamethasone only moderately, but not significantly, induced femoral and tibia trabecular bone loss in *Tau*^*−/−*^ mice (Fig. 2g, h). The bone microarchitecture parameters such as bone volume/total volume (BV/TV), trabecular thickness (Tb.Th), and trabecular number (Tb.N) are influenced by the cumulative activity of both osteoclasts and osteoblasts over a longer period, therefore, the dexamethasone-induced reduction in osteoblast numbers in *Tau*^*−/−*^ mice might not be sufficient to cause significant changes in the bone volume parameters within the given experimental timeframe. This result suggests that high-dose dexamethasone increased bone resorption through Tau, outweighing its decreased bone formation through GR, at least under our current setting. In agreement with the notion that the inflammatory process in RA is associated with bone loss independent of GCs,^33^ CIA mice with various genetic background exhibited reduced bone mass. In addition, exaggerated inflammation in *GR*^*−/−*^ CIA mice provoked more bone loss than that in WT and *Tau*^*−/−*^ CIA mice (Fig. 2g, h). Collectively, these data demonstrate that treatment of CIA mice with dexamethasone resembles a state of GIO with inflammatory arthritis. Additionally, dexamethasone exerts its anti-inflammatory action via GR and its adverse effect, i.e., bone resorption, via Tau.

### Tau is required for high-dose dexamethasone-induced bone resorption in GIO model without inflammation

**Fig. 3:**
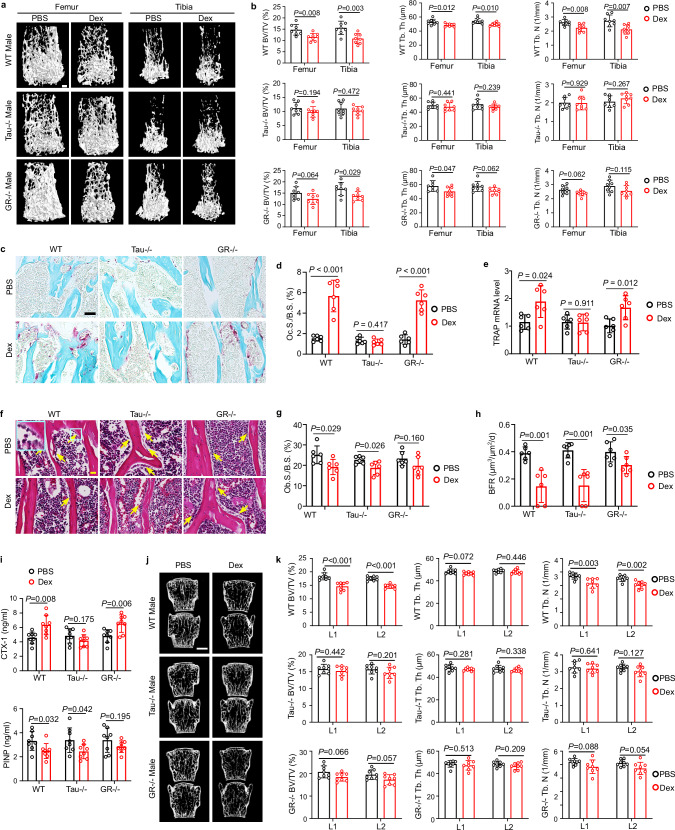
Tau is required for GIO. **a** Representative reconstructed 3D micro-CT images of femoral and tibia trabecular bone from WT, *Tau*^*−/−*^ and *GR*^*−/−*^ male mice with or without GIO. Scale bar = 250 µm. **b** Quantification of trabecular parameters including BV/TV, Tb.Th and Tb.N in WT, *Tau*^*−/−*^ and *GR*^*−/−*^ male mice with or without GIO (*n* = 8 mice for each group). **c**, **d** Representative TRAP staining image (**c**), and quantification of TRAP^+^ Oc.S./B.S. (**d**) of femoral distal metaphysis of WT, *Tau*^*−/−*^ and *GR*^*−/−*^ male mice in the same experiment (*n* = 6 mice for each group). Scale bar = 50 µm. **e** *TRAP* mRNA level in WT, *Tau*^*−/−*^ and *GR*^*−/−*^ femur after treated with dexamethasone for 5 weeks (*n* = 6 mice for each group). **f** Representative H&E staining images of femur showing osteoblasts (yellow arrows) on the trabecular bone. The area defined by blue rectangle on the representative image is enlarged as inset. Scale bar = 20 µm. **g** Quantification of Ob.S./B.S. in the indicated mice. **h** Bone formation rate (BFR) determined by calcein labeling in WT, *Tau*^*−/−*^, *GR*^*−/−*^ male mice after treated with or without dexamethasone for 5 weeks (*n* = 6 mice for each group). **i** Levels of CTX-1 and PINP in the indicated male mice sera, assayed with ELISA (*n* = 8 mice for each group). **j**, **k** Representative reconstructed 3D micro-CT images (**j**) and quantification (**k**) of trabecular bone of the L1 and L2 vertebra of WT, *Tau*^*−/−*^ and *GR*^*−/−*^ male mice treated with or without dexamethasone for 5 weeks (*n* = 6 mice for each group). Scale bar = 1 mm in **j**. Data are means ± SD. *P* values are calculated by two-tailed unpaired Student’s *t*-test (**b**, **d**, **e**, **g–i**, **k**).

To distinguish the contributions of Tau and GR to dexamethasone-induced bone loss in the absence of inflammation, we established a common GIO model with male mice treated with 10 mg/kg body weight dexamethasone for a total of 5 weeks. Dexamethasone significantly reduced bone mass in WT mice, evidenced by significant lower femoral and tibia trabecular bone volume fraction, trabecular thickness and trabecular number. This reduction was less pronounced and partially lost in dexamethasone-treated *GR*^*−/−*^ mice, while it was largely lost in dexamethasone-treated *Tau*^*−/−*^ mice (Fig. 3a, b). In line with the observations in dexamethasone-treated CIA mice, dexamethasone dramatically increased osteoclasts in femoral trabecular bone of WT and *GR*^*−/−*^, but not those of *Tau*^*−/−*^ mice (Fig. 3c, d). Consistent with these findings, Tau deficiency abolished dexamethasone-upregulated TRAP observed in the femur of WT and *GR*^*−/−*^ mice (Fig. 3e). In contrast, dexamethasone significantly decreased osteoblast surface and bone formation rates in WT and *Tau*^*−/−*^ mice, but not in *GR*^*−/−*^ mice (Fig. 3f–h; Supplementary information, Fig. S3f, g), implying that GR is needed for dexamethasone-mediated inhibition of bone formation. In addition, dexamethasone significantly increased serum levels of bone resorption marker CTX-1 in WT and *GR*^*−/−*^ mice (Fig. 3i). In contrast, in *Tau*^*−/−*^ mice, serum levels of CTX-1 were not increased after dexamethasone treatment (Fig. 3i). Meanwhile, dexamethasone decreased serum levels of bone formation marker PINP in both WT and *Tau*^*−/−*^ mice, but not in *GR*^*−/−*^ mice (Fig. 3i).

Since GIO predominantly affects the bone regions composed largely of cancellous bone, such as vertebrae and femur,^34^ we also evaluated the effects of Tau deficiency on bone quality in vertebrae. Similar to our observations in femur and tibia, Tau deficiency also largely ablated dexamethasone-induced bone loss in vertebrae, whereas dexamethasone led to a significant reduction in trabecular bone volume fraction, trabecular number in WT vertebrae (Fig. 3j, k). In *GR*^*−/−*^ mice, dexamethasone tended to reduce trabecular bone volume fraction and trabecular number; however, this reduction did not reach statistical significance (Fig. 3j, k).

To examine potential sex differences, we also repeated these assays with female mouse models and found that the findings with μCT, dual energy X-ray absorption (DEXA), TRAP, osteoblast surface analysis and ELISA in male mice were recapitulated in female mice (Supplementary information, Figs. S2g, i, 3a–h), indicating dexamethasone-caused Tau-dependent bone resorption is sex independent.

In brief, GIO primarily arises from Tau-mediated enhancement of osteoclast activity and subsequent bone resorption in response to high-dose dexamethasone. Additionally, GR-dependent inhibition of bone formation also contributes to the development of GIO.

### Tau is required for high-dose dexamethasone-mediated osteoclastogenesis in vitro

At low physiological concentrations, endogenous GCs are essential for mesenchymal cell differentiation and function and relevant signaling is primarily mediated through GR.^35,36^ However, at high pharmacological concentrations, long-term use of GCs leads to the development of GIO, the most common side effect conferred by GCs.^37^ In human subjects, GIO is characterized by a rapid increase in bone resorption followed by impaired bone formation.^38^ As such, we then asked whether Tau was also involved in high-dose dexamethasone-mediated perturbations in osteoblastogenesis and osteoclastogenesis in vitro.

**Fig. 4:**
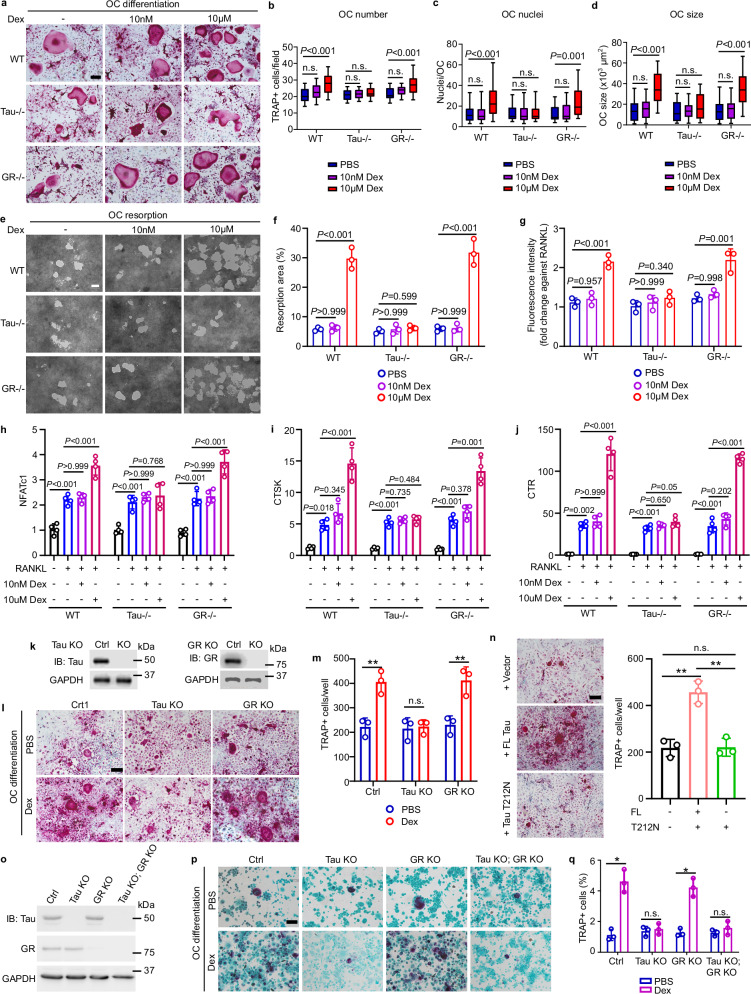
Tau is required for high-dose dexamethasone-mediated enhancement of osteoclastogenesis in vitro. **a** Representative bright-field images of TRAP-positive bone marrow macrophage-derived osteoclasts in vitro. Bone marrow-derived macrophages isolated from WT, *Tau*^*−/−*^ and *GR*^*−/−*^ mice are differentiated with 20 ng/mL M-CSF and 50 ng/mL RANKL supplemented with low (10 nM) or high (10 µM) dose of dexamethasone for 7 days. Sale bar = 100 µm. **b** Quantification of number of TRAP^+^ multinuclear osteoclast shown in **a** (*n* = 20, number per field of view, from three mice per age group). **c**, **d** Quantification of nuclei number (**c**) and size (**d**) per osteoclast shown in **a** (*n* = 25 per group). **e**–**g** Representative microscopic images of resorption pit (**e**), quantification of the resorption pit areas (**f**) and fluorescence intensity released into media (**g**), assayed with the osteoclasts derived from WT, *Tau*^*−/−*^ and *GR*^*−/−*^ mouse bone marrow macrophages cultured with 20 ng/mL M-CSF and 50 ng/mL RANKL supplemented with low or high dose of dexamethasone for 7 days (*n* = 3). **h**–**j** Gene expression levels of osteoclast differentiation markers *NFATc1* (following 3 days of differentiation), *CTSK* and *CTR* (following 5 days of differentiation) determined by qRT-PCR. Bone marrow-derived macrophages isolated from WT, *Tau*^*−/−*^ and *GR*^*−/−*^ mice are differentiated with 20 ng/mL M-CSF and 50 ng/ml RANKL supplemented with low or high dose of dexamethasone (*n* = 4). **k** *Tau* knockout and *GR* knockout Raw264.7 macrophage are generated by CRISPR-Cas9 technique. The knockout efficiency of *Tau* and *GR* is determined using immunoblotting. **l**, **m** Representative bright-field images (**l**) and corresponding quantification (**m**) of TRAP-positive multinuclear osteoclasts differentiated from Raw264.7 macrophages treated with 50 ng/mL RANKL in the presence of 10 µM dexamethasone for 5 days (*n* = 3). Scale bar = 100 µm. **n** Representative bright-field images and corresponding quantification of TRAP-positive multinuclear osteoclasts differentiated from full-length (FL) or Tau T212N point mutant transfected *Tau*^*−/−*^ Raw264.7 macrophages treated with 50 ng/mL RANKL and 10 µM dexamethasone for 5 days (*n* = 3). Scale bar = 100 µm. **o** Immunoblotting to demonstrate knockout efficiency in *Tau*^*−/−*^, *GR*^*−/−*^ and *Tau*^*−/−*^; *GR*^*−/−*^ THP-1 human monocytes generated by CRISPR-Cas9. **p**, **q** Representative bright-field images (**p**) and corresponding quantification (**q**) of TRAP-positive multinuclear osteoclast differentiated from THP-1-derived macrophages with 50 ng/mL RANKL in the presence of 10 µM dexamethasone for 14 days (*n* = 3). Scale bar = 100 µm. Data are means ± SD, except **b**–**d**, which show box and whisker plots with center line as median, box extending from 25^th^ to 75^th^ percentile and whiskers extending from minimum to maximum values. *P* values are calculated by one-way ANOVA with Bonferroni post-hoc test (**b**–**d**, **f**–**j**, **n**) and two-tailed unpaired Student’s *t*-test (**m**, **q**). ns, not significant; * *P* < 0.05; **P < 0.01.

In our experimental setting, both low- and high-dose dexamethasone did not affect osteoclast and osteoblast apoptosis obtained from the bone marrow of WT, *Tau*^*−/−*^^30^ and *GR*^*−/−*^ mice (Supplementary information, Fig. S4a, b). High-dose dexamethasone significantly enhanced RANKL- and M-CSF-primed osteoclastogenesis of bone marrow mononuclear cells isolated from WT and *GR*^*−/−*^ mice, but not those from *Tau*^*−/−*^ mice (Fig. 4a–g). qRT-PCR analysis further disclosed that high-dose dexamethasone significantly increased the expressions of osteoclast differentiation and bone resorption markers *NFATc1*, *Cathepsin K* (*CTSK*) and *calcitonin receptor* (*CTR*), in bone marrow cells isolated from WT and *GR*^*−/−*^ mice, but not in those cells from *Tau*^*−/−*^ mice (Fig. 4h–j), indicating that high-dose dexamethasone-enhanced osteoclastogenesis depended on Tau. Consistently, knockout of *Tau* did, while knockout of *GR* did not, ablate dexamethasone-enhanced RANKL-primed osteoclastogenesis of mouse Raw264.7 macrophages (Fig. 4k–m). Thr212, located within the proline-rich region of Tau, was the most critical amino acid for interaction with high-dose dexamethasone (Supplementary information, Fig. S1). Re-introduction of Tau with point mutation Thr212Asn into *Tau* knockout Raw264.7 cells revealed that this mutation failed to restore high-dose dexamethasone-mediated osteoclastogenesis. This finding pinpointed the importance of the proline-rich region in dexamethasone-enhanced osteoclastogenesis (Fig. 4n). Additionally, knockout of *Tau* or *GR* in human THP-1 monocytes closely recapitulated the results observed in primary mouse bone marrow derived macrophage (BMDM) and Raw264.7 (Fig. 4o–q). Notably, in contrast to high-dose dexamethasone-enhanced osteoclastogenesis through Tau, we observed that high-dose dexamethasone inhibited osteoblastogenesis largely through GR (Supplementary information, Fig. S4c–h). Taken together, these data indicate that Tau is required for high-dose dexamethasone-mediated osteoclastogenesis.

### TTBK1 phosphorylates Tau at residue Ser422 during high-dose dexamethasone-enhanced osteoclastogenesis

**Fig. 5:**
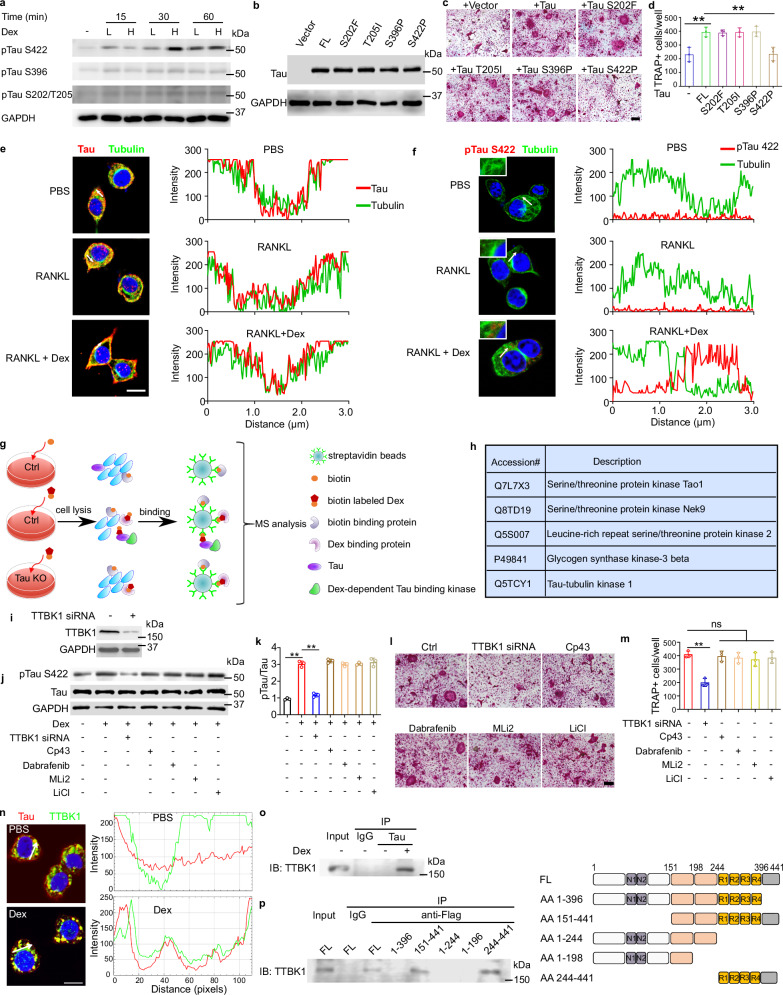
Tau Ser422 phosphorylation by high-dose dexamethasone through TTBK1 is required for dexamethasone-enhanced osteoclastogenesis. **a** Immunoblotting of pTau S422, S396 and S202/T205 in Raw264.7 cells treated with low (L, 10 nM) or high (H, 10 µM) dose of dexamethasone along with 50 ng/mL RANKL for the indicated time. GAPDH is used as a loading control. Representative images from duplicate results are shown. **b** Immunoblotting of Tau in *Tau* knockout Raw264.7 cells transfected with empty vector, full length (FL) or Tau with serial point mutations. Representative images of duplicate results are shown. **c**, **d** Representative bright-field images (**c**) and corresponding quantification (**d**) of TRAP-positive multinuclear osteoclasts differentiated from FL- or Tau with serial point mutations- transfected *Tau*^*−/−*^ Raw264.7 macrophage treated with 50 ng/mL RANKL and 10 µM dexamethasone for 5 days. Scale bar = 100 µm. **e** Confocal images of Raw264.7 cells labeled with antibodies for Tau and tubulin (left panel), and corresponding fluorescence signal intensity plots of Tau (red) and tubulin (green) vesicles (right panel). Cells are treated with or without 50 ng/mL RANKL and 10 µM dexamethasone. Arrows indicate regions in each cell where fluorescent signal intensity plots are obtained. **f** Confocal images of Raw264.7 cells labeled with antibodies for p-Tau Ser422 and tubulin (left panel), and corresponding fluorescence signal intensity plots of p-Tau Ser422 (red) and tubulin (green) vesicles (right panel). Cells are treated with or without 50 ng/mL RANKL and 10 µM dexamethasone. Arrows indicate regions in each cell where fluorescent signal intensity plots are obtained. **g** Experimental design and principle used to identify the potential kinases that are involved in dexamethasone-dependent p-Tau Ser422. **h** Summary of the potential kinases responsible for dexamethasone-dependent activation of Tau. **i** Knockdown efficiency of TTBK1 in Raw264.7 cells, measured by western blot (*n* = 3). **j** Immunoblotting of dexamethasone-activated p-Tau Ser422 in Raw264.7 cells transfected with TTBK1 siRNA or treated with different kinase inhibitors, as indicated. **k** Densitometry analysis of immunoblotting results shown in **j**. **l**, **m** Representative bright-field images (**l**) and corresponding quantification (**m**) of TRAP-positive multinuclear osteoclast differentiated from Raw264.7 macrophages, which are transfected with TTBK1 siRNA or treated with different kinase inhibitor, followed by stimulating with 50 ng/mL RANKL and 10 µM dexamethasone for 5 days. Scale bar = 100 µm. **n** Confocal images of vehicle- or 10 µM dexamethasone-treated Raw264.7 cells labeled with antibodies for Tau and TTBK1 (left panel), and corresponding fluorescence signal intensity plots of Tau (red) and TTBK1 (green) vesicles (right panel). Arrows indicate regions in each cell where fluorescent signal intensity plots are obtained. **o** IP from 10 µM dexamethasone-treated Raw264.7 cells with anti-Tau antibody, and detection of TTBK1 by immunoblotting. Representative image of duplicate results is shown. **p** IP from 10 µM dexamethasone-treated Raw264.7 cells transfected with FLAG-tagged FL Tau or Tau with serial deletion mutations using anti-FLAG M2 resins, and detection of TTBK1 by immunoblotting (*n* = 3). Data are means ± SD. *P* values are calculated by one-way ANOVA with Bonferroni post-hoc test (**d**, **k**, **m**).

Phosphorylation is one of the most common post-translational modifications of Tau and may occur at many serine and threonine residues by a variety of kinases under physiological and pathological conditions.^39,40^ To assess whether high-dose dexamethasone was able to induce the phosphorylation of Tau, the total protein fraction of Raw264.7 cells treated with low- or high-dose dexamethasone was examined by immunoblotting with antibodies recognizing Tau phosphorylated at residues Ser202/Thr205, Ser396 and Ser422, the well characterized phosphorylation sites in other diseases. High-dose dexamethasone preferentially phosphorylated Tau at residue Ser422 (Fig. 5a). Re-introduction of Tau with point mutations of these phosphorylated sites into *Tau* knockout Raw264.7 cells revealed that the point mutation on Tau Ser422 failed to restore high-dose dexamethasone-mediated osteoclastogenesis, reinforcing the finding that Tau Ser422 is the critical phosphorylation site implicated in this process (Fig. 5b–d).

Tau is a microtubule-binding protein and associated with dynamic regulation of the cytoskeleton through direct binding to tubulin.^41^ Using confocal microscopy and fluorescent signal intensity plots of total Tau, Tau phosphorylated at Ser422 (p-Tau Ser422) and tubulin, we found that high-dose dexamethasone activated Tau phosphorylation at Ser422 and this phosphorylated Tau did not co-localize with tubulin, although total Tau well co-localized with tubulin, particularly under the untreated conditions (Fig. 5e, f). Additionally, unlike GR which is primarily located in the cytoplasm and translocated to the nucleus upon binding to GCs to regulate gene transcription, both Tau and p-Tau remained primarily in the cytoplasm in response to high-dose dexamethasone. These findings indicate that p-Tau Ser422, which accounts for a very small percentage of total Tau, was not associated with microtubules and did not translocate into nucleus in mediating high-dose dexamethasone-induced osteoclastogenesis.

To identify the kinase responsible for Tau phosphorylation at Ser422, we performed biochemical co-purification coupled with proteomics analysis with control and *Tau* knockout Raw264.7 cells treated with high-dose biotin-labeled dexamethasone, and identified 5 kinases that were recruited to Tau by high-dose dexamethasone (Fig. 5g, h). We next knocked down TTBK1 with its specific siRNA and inhibited 4 other kinases with their specific pharmacological inhibitors to determine the potential involvements of these kinases in high-dose dexamethasone-enhanced osteoclastogenesis. These assays led to the isolation of TTBK1, the kinase reported to phosphorylate Tau at residue Ser422,^42^ as the only critical kinase responsible for phosphorylation of Tau Ser422 in mediation of high-dose dexamethasone-enhanced osteoclastogenesis (Fig. 5i–m). Imaging with confocal microscopy revealed that high-dose dexamethasone induced the co-localization of TTBK1 with Tau (Fig. 5n). Additionally, co-immunoprecipitation (IP) assays revealed dexamethasone-dependent interaction of TTBK1 with full length Tau. Furthermore, domain mapping experiment with serial Tau deletion mutants showed that amino acids 396–441, which constitute Tau C-terminal region, were responsible for its binding to TTBK1 (Fig. 5o, p). Taken together, these results indicate that high-dose dexamethasone triggered the recruitment of TTBK1 kinase to Tau, leading to the phosphorylation of Tau at residue Ser422, thereby enhancing osteoclastogenesis.

### Activated p-Tau Ser422 promotes the processing of NF-κB1 p105 into p50 and enhances its nuclear translocation during high-dose dexamethasone-enhanced osteoclastogenesis

**Fig. 6:**
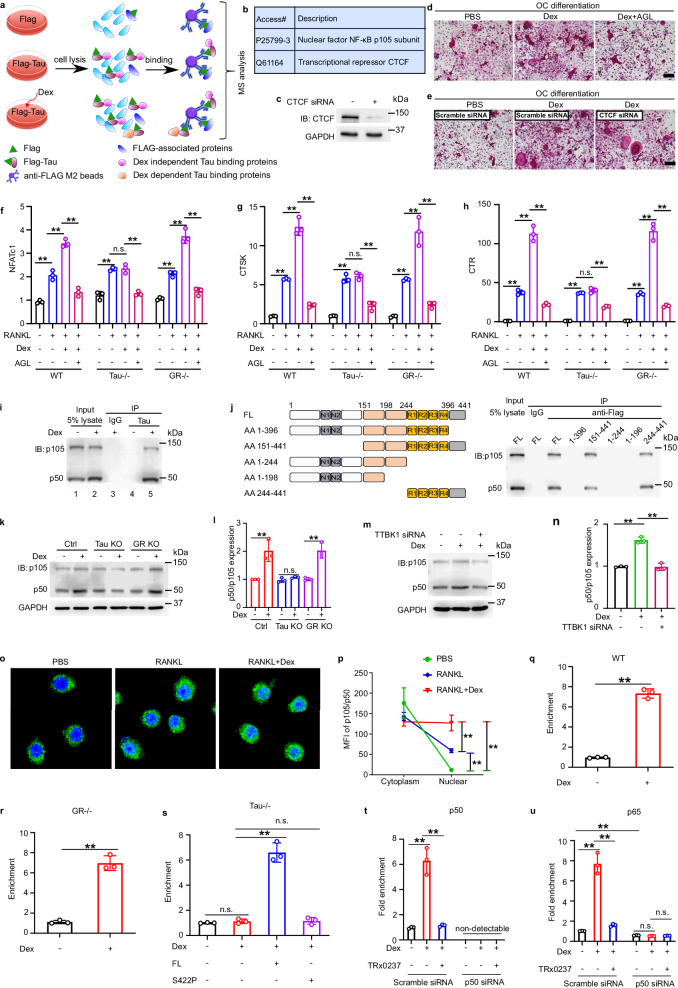
p105/p50 is a downstream mediator of phosphorylated Tau in mediation of high-dose dexamethasone-enhanced osteoclastogenesis. **a** Schematic for application of biochemical co-purification and mass spectrometry approaches to screen the transcriptional factors recruited to activated Tau by high-dose dexamethasone. Raw264.7 cells transfected with FLAG or FLAG-tagged Tau are treated with or without 10 µM dexamethasone for 30 min, followed by precipitation with anti-FLAG M2 resins. **b** Summary of the identified transcriptional factors recruited to Tau in 10 µM dexamethasone-treated Raw264.7 cells. **c** Knockdown efficiency of CTCF in Raw264.7 cells, measured by immunoblotting. **d**, **e** Representative bright-field images of TRAP-positive multinuclear osteoclast differentiated from Raw264.7 macrophages treated with 50 ng/mL RANKL and 10 µM dexamethasone for 5 days. Raw264.7 cells are treated with andrographolide (AGL, p50 inhibitor) or transfected with CTCF siRNA before differentiating into osteoclasts. Scale bar = 100 µm. **f**–**h** Gene expression levels of osteoclast differentiation markers *NFATc1* (following 3 days of differentiation), *CTSK* and *CTR* (following 5 days of differentiation) determined by qRT-PCR. Bone marrow-derived macrophage isolated from WT, *Tau*^*−/−*^ and *GR*^*−/−*^ mice are differentiated with 20 ng/mL M-CSF and 50 ng/mL RANKL supplemented with low or high dose of dexamethasone (*n* = 3). **i** IP from 10 µM dexamethasone-treated Raw264.7 cells with anti-Tau antibody, and detection of p105/p50 by immunoblotting. Representative image of duplicate results is shown. **j** IP from 10 µM dexamethasone-treated Raw264.7 cells transfected with FLAG-tagged FL or serial deletion mutations of Tau with anti-FLAG M2 resins, and detection of p105/p50 by immunoblotting. **k**, **l** Immunoblotting analysis (**k**) and quantification (**l**) of relative levels of p105 and p50 in control, *Tau*^*−/−*^ and *GR*^*−/−*^ Raw264.7 cells after 10 µM dexamethasone stimulation (*n* = 3). **m**, **n** Immunoblotting analysis (**m**) and quantification (**n**) of relative levels of p105 and p50 in TTBK1 siRNA-transfected Raw264.7 cells after 10 µM dexamethasone stimulation. **o**, **p** Confocal images of Raw264.7 cells labeled with antibodies for p105/p50 (**o**), and relative fluorescence intensity of p105/p50 in cytoplasm and nuclear are shown (**p**) (*n* = 3 biological replicates). **q**, **r** ChIP-qPCR assay of p50 in the *NFATc1* promoter in WT (**q**) or *GR*^*−/−*^ (**r**) Raw264.7 cells treated with 50 ng/mL RANKL for 1 h. **s** ChIP-qPCR assay of p50 in the *NFATc1* promoter in *Tau*^*−/−*^ Raw264.7 cells transfected with or without FL Tau or Tau S422P mutant followed by treatment with 50 ng/mL RANKL for 1 h. **t**, **u** ChIP-qPCR assay of p50 (**t**) and p65 (**u**) in the *NFATc1* promoter in control or p50 siRNA knockdown Raw264.7 cell treated with RANKL in the presence or absence of Trx0237 for 1 h. Data are means ± SD. *P* values are calculated by one-way ANOVA with Bonferroni post-hoc test  (**f**–**h**, **n**, **s**–**u**), two-way ANOVA with Bonferroni post-hoc test (**p**) and two-tailed unpaired Student’s *t*-test (**l**, **q**, **r**). n.s., not significant; ** *P* < 0.01.

To identify the transcription factor(s) downstream of p-Tau Ser422 that may mediate high-dose dexamethasone-induced osteoclastogenesis, we performed another biochemical co-purification coupled with proteomics analysis using *Tau* knockout Raw264.7 cells transfected with or without FLAG-tagged Tau (Fig. 6a). Five transcription factors were detected in the immunoprecipitated complexes of Tau in response to high-dose dexamethasone treatment (Supplementary information, Table S1). Based on their known function, we mainly focused on two of them, NF-κB p105 and CTCF (Fig. 6b). NF-κB p50 is cleaved from its precursor p105 through a proteasome-mediated processing.^43^ We thus inhibited NF-κB p50 activity with andrographolide^44^ or knocked down CTCF with its specific siRNA, and found that high-dose dexamethasone-mediated osteoclastogenesis was blocked once NF-κB p50 activity was inhibited, and was not affected by knockdown of CTCF (Fig. 6c, d). qRT-PCR analysis demonstrated that inhibiting p50 activity abolished high-dose dexamethasone-enhanced expression of osteoclastogenesis markers including *NFATc1*, *CTSK* and *CTR*, and decreased RANKL-induced osteoclastogenesis (Fig. 6f–h). In addition, co-IP demonstrated dexamethasone-dependent interaction between Tau and p105/p50. Furthermore, deletion of amino acids 396–441 of the C-terminal region in Tau impaired the association between Tau and p105/p50 (Fig. 6i, j). Of note, high-dose dexamethasone also stimulated the processing of p105 into p50 (Fig. 6k, compare lanes 1 and 2), and deletion of Tau or knockdown of TTBK1 blocked this dexamethasone-dependent processing of p105 into p50 (Fig. 6k–n). In agreement with a previous report that NF-κB p50 preferentially formed heterodimers with p65 to translocate into nucleus and induce osteoclastogenesis in response to RANKL,^45^ stimulation of Raw264.7 cells with RANKL primed the nuclear translocation of p50 and dexamethasone further enhanced this process (Fig. 6o, p). Additionally, chromatin IP (ChIP)-qPCR was conducted to analyze p50 binding to the promoter of *NFATc1*, the critical transcriptional factor essential for osteoclastogenesis, in WT (Raw264.7), *GR*^*−/−*^, *Tau*^*−/−*^ and *Tau*^*−/−*^ cells transfected with either full-length Tau or the phosphosite point mutant Tau S422P. Results from these ChIP-qPCR assays demonstrated an increase in p50 binding to the *NFATc1* promoter in high-dose dexamethasone-treated WT and *GR*^*−/−*^ cells, but not in *Tau*^*−/−*^ cells (Fig. 6q–s). However, re-expression of full-length Tau, but not the phosphosite point mutant Tau S422P, in *Tau*^*−/−*^ cells restored high-dose dexamethasone-mediated enhanced binding of p50 to the *NFATc1* promoter (Fig. 6s). Collectively, high-dose dexamethasone phosphorylates Tau to activate transcription factor p50 independent of GR, in turn, leading to enhanced osteoclastogenesis.

### Discovery of TRx0237 as a Tau inhibitor that inhibits dexamethasone-induced Tau phosphorylation at Ser422 and osteoclastogenesis

Since phosphorylation of Tau at Ser422 was a critical molecular event required to mediate dexamethasone-dependent osteoclastogenesis, we explored the possibility of inhibiting Tau Ser422 phosphorylation as a therapeutic target for GIO. For this purpose, we screened an FDA-approved drug library including 958 drugs using an ELISA-based high throughput method (Supplementary information, Fig. S5a), and identified two potential drugs, raloxifene and TRx0237, that could inhibit dexamethasone-induced Tau phosphorylation at Ser422 (Supplementary information, Fig. S5b, c). MB and its derivatives are known Tau aggregation inhibitors that hold promising preclinical results for Alzheimer’s disease^46,47^ and have advanced to clinical trials,^48^ although none have been approved for clinical use. TRx0237 is a derivative of MB in a stabilized and reduced form^47^ and failed to show clinical efficacy in Phase III clinical trials in Alzheimer’s disease patients (NCT01689246 and NCT01689233).^49^ Here, we found that MB, TRx0237, and raloxifene significantly inhibited dexamethasone-dependent activation of Tau phosphorylation at Ser422 (Supplementary information, Fig. S5d, e). Secondary screening of these 3 drugs using TRAP staining revealed that only MB and TRx0237, but not raloxifene, blocked dexamethasone-dependent osteoclastogenesis in a Tau-dependent manner (Supplementary information, Fig. S5f–j). Moreover, pit assay confirmed that both MB and Trx0237 ablated dexamethasone-mediated bone resorption in a Tau-dependent manner (Supplementary information, Fig. S5k–m). Raloxifene is a selective estrogen receptor modulator that is an FDA approved drug for treating postmenopausal osteoporosis.^50^ Of note, raloxifene failed to inhibit dexamethasone-dependent osteoclastogenesis despite abolishing dexamethasone-mediated activation of Tau phosphorylation at Ser422, suggesting that additional post-translational modifications including phosphorylation at other residues beyond Ser422, ubiquitination, acetylation, methylation, oxidation and glycation, which were reported to play key roles in Tau functions in other diseases,^51^ may also be involved in dexamethasone-enhanced osteoclastogenesis. Further, we conducted ChIP-qPCR assay to determine the impact of TRx0237 on nuclear NF-κB activity. The results revealed that TRx0237 significantly inhibited p50 and co-regulator p65 recruitment to the *NFATc1* promoter. This inhibition was not observed in p50-knockdown Raw264.7 cells (Fig. 6t, u), confirming that increased nuclear p50 activity is responsible for the high-dose dexamethasone-enhanced osteoclast activity. In summary, TRx0237 holds promise as a potential drug to antagonize dexamethasone-induced Tau-dependent osteoclastogenesis.

### TRx0237 is therapeutic against GC-induced osteoporosis through inhibition of Tau phosphorylation at Ser422

We then asked whether TRx0237 is therapeutic against GIO in vivo. Indeed, TRx0237 treatment was associated with increased trabecular BV/TV and trabecular number in long bone and vertebra of dexamethasone-treated WT and *GR*^*−/−*^ mice, despite having no effects on trabecular bone thickness in long bone (Supplementary information, Figs. S6a–e, S7a, b). Dexamethasone did not cause obvious bone loss in *Tau*^*−/−*^ mice, and as expected, TRx0237 did not exert any effects on bone quality in terms of trabecular BV/TV, thickness and number (Supplementary information, Fig. S6b–e). Histology analysis revealed that TRx0237 abolished high-dose dexamethasone-induced osteoclast activity, but did not affect osteoblast surface values in both long bones and vertebrae of WT and *GR*^*−/−*^ mice (Supplementary information, Figs. S6f–h, S7c–e). Consistently, TRx0237 blocked the elevated serum levels of CTX-1 in dexamethasone-treated WT and *GR*^*−/−*^ mice, but did not affect serum levels of the bone formation marker PINP (Supplementary information, Fig. S7f–h). Consistent with our observation that TRx0237 abolished dexamethasone-mediated osteoclast activity through inhibition of p-Tau Ser422 in vitro, TRx0237 markedly reduced Tau phosphorylation at Ser422 in bone tissues of WT and *GR*^*−/−*^ GIO mice (Supplementary information, Fig. S7i). Taken together, we successfully isolated TRx0237 from an FDA-approved drug library that protected against GIO in vivo. Notably, in *GR*^*−/−*^ mice, although dexamethasone did not significantly reduce trabecular bone volume and trabecular number in both long bone and vertebra, the Tau inhibitor TRx0237 still significantly mitigated bone loss. This finding further underscores the important role of Tau in mediating bone resorption in GIO.

Next, we sought to explore the potential dose-dependent effects and determine the optimum dosage of TRx0237 with WT GIO mouse model. DEXA scanning revealed that all three dosages of TRx0237 employed were able to effectively protect against dexamethasone-induced bone loss (Supplementary information, Fig. S8a). μCT analysis of trabecular bone at femur and tibia also demonstrated that all dosages of TRx0237 increased bone volume fraction, and trabecular number in GIO mice without affecting trabecular thickness (Supplementary information, Fig. S8b–e). Nonetheless, even lowest dose of TRx0237 effectively decreased dexamethasone-induced serum levels of bone resorption marker CTX1, despite having no effect on bone formation marker PINP (Supplementary information, Fig. S8f). In addition, the lowest dose of TRx0237 blocked dexamethasone-induced osteoclast activity (Supplementary information, Fig. S8g, h). Consistent with our observation that TRx-237 abolished dexamethasone-mediated osteoclast activity through inhibiting Tau phosphorylation at Ser422 in vitro, all three doses of TRx0237 markedly reduced Tau phosphorylation at Ser422 in bone tissue of GIO mice as compared to that of GIO mice without treatment. Notably, highest dose of TRx0237 reduced dexamethasone-activated Tau phosphorylation at Ser422 to non-dexamethasone-treated level (Supplementary information, Fig. S8i, j). Taken together, all these dosages employed in the current study effectively ablated dexamethasone-caused bone loss, and further study is needed to determine the optimal lowest dosage of TRx0237.

### Combinatorial treatment with dexamethasone and TRx0237 overcomes dexamethasone-induced bone loss in treating inflammatory arthritis

**Fig. 7:**
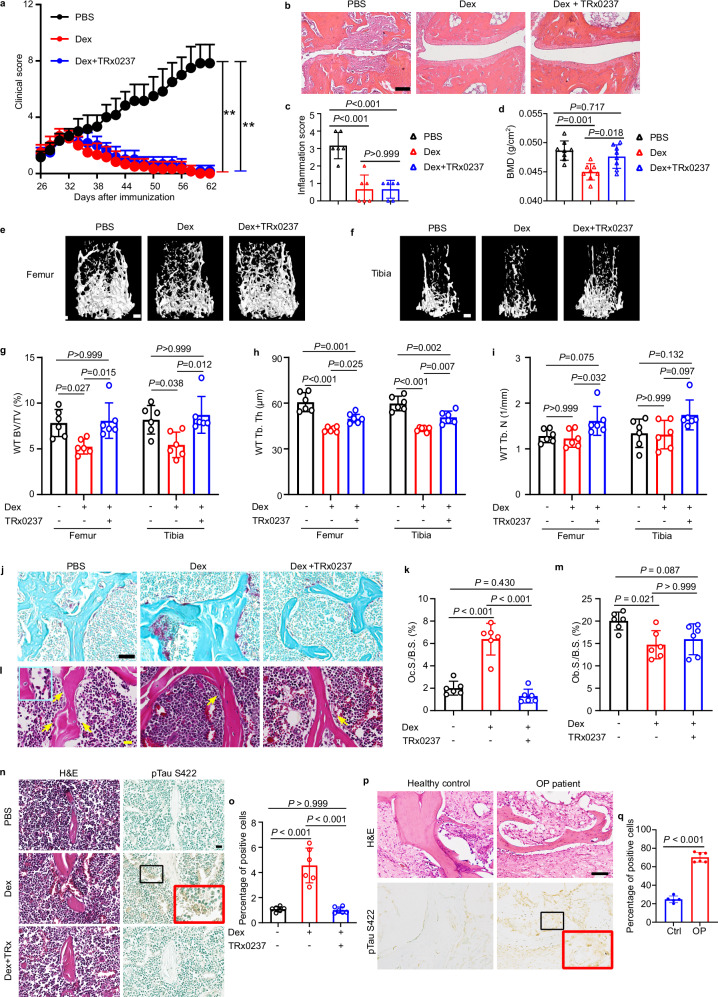
Combinatorial treatment with dexamethasone and TRx0237 enables anti-inflammatory action while prevents dexamethasone-induced bone loss in CIA. **a** Clinical arthritis scores of WT CIA mice treated with or without dexamethasone in the presence or absence of TRx0237, as indicated, for 5 weeks (*n* = 6 mice for each group). **b**, **c** Representative images of H&E staining (**b**), and quantification of histomorphometric analysis of synovial inflammation (**c**) of ankle joints from indicated mice (*n* = 6 mice for each group). Scale bar = 100 µm in **b**. **d** Whole body bone mineral density in WT male mice with CIA treated with or without dexamethasone in the presence or absence of TRx0237, as indicated, for 5 weeks, measured by DEXA scanning (*n* = 8 mice for each group). **e**, **f** Representative reconstructed 3D micro-CT images, of trabecular bone of femur (**e**) and tibia (**f**) of WT male CIA mice with indicated treatment. Scale bar = 250 µm. **g**–**i** Quantification of femur and tibia trabecular BV/TV (**g**), Tb.Th (**h**) and Tb.N (**i**) in the same experiment (*n* = 6 mice for each group). **j**, **k** Representative TRAP staining image (**j**), and quantification of TRAP^+^ osteoclast surface per bone surface (Oc.S./B.S.) (**k**) of the femur distal metaphysis of WT male mice with CIA in the same experiment (*n* = 6 mice for each group). Scale bar = 50 µm. **l**, **m** Representative H&E-stained images of femur showing osteoblasts (yellow arrows) on the trabecular bone (**l**). The area defined by blue rectangle on the representative image is enlarged as inset. Scale bar = 20 µm. Quantification of osteoblast surface per bone surface (Ob.S./B.S.) in the indicated mice (**m**). **n**, **o** H&E and immunohistochemistry staining (**n**), and corresponding quantification (**o**) of p-Tau S422-positive cells in femur of WT male mice with CIA in the same experiment (*n* = 6 mice for each group). The areas defined by black rectangle on each representative image are enlarged as insets. Scale bar = 20 µm. **p**, **q** H&E and immunohistochemistry staining (**p**), and corresponding quantification (**q**) of p-Tau Ser422 in human bone tissue of healthy controls (*n* = 5) and osteoporosis patients (*n* = 6). The areas defined by black rectangle on each representative image are enlarged as insets, scale bar = 100 µm. Data are means ± SD, *P*-values are calculated by two-way ANOVA with Bonferroni post-hoc test (**a**), one-way ANOVA with Bonferroni post-hoc test (**c**, **d**, **g**–**i**, **k**, **m**, **o**) and two-tailed unpaired Student’s *t*-test (**q**).

RA is a chronic inflammatory arthritis affecting 0.5%–1% adults globally.^52^ Despite adverse effects such as GIO, GCs continue to be an important component of RA therapy in clinic due to their broad-spectrum anti-inflammatory effects.^53–55^ Here, we used CIA mice treated with dexamethasone to determine whether combinational treatment with anti-inflammatory dexamethasone and anti-GIO TRx0237 in CIA mice could overcome dexamethasone-induced bone loss while retaining its anti-inflammatory action. Combinational treatment elicited an anti-inflammatory effect un-distinguishable from that observed with dexamethasone monotherapy in CIA mice (Fig. 7a–c). Of note, in line with the observation that genetic ablation of *Tau* prevents GIO, pharmacological administration of Tau inhibitor TRx0237 in dexamethasone-treated CIA mice effectively protected against dexamethasone-induced bone loss through inhibiting osteoclasts, without affecting osteoblasts (Fig. 7d–m; Supplementary information, Fig. S2j, k). Immunohistochemistry staining revealed that dexamethasone-dependent Tau phosphorylation at Ser422 was also abolished by TRx0237 in CIA mice (Fig. 7n, o), further supporting the identification of p-Tau Ser422 as a therapeutic target in osteoporosis. Collectively, these data indicate that combinatorial treatment with dexamethasone and TRx0237 keeps the anti-inflammatory action while avoids the adverse effect of dexamethasone in treating inflammatory arthritis.

We also examined the association between p-Tau Ser422 and bone loss in bone tissues from healthy controls and patients with osteoporosis. Significantly higher p-Tau Ser422 levels were observed in bone tissues from osteoporosis patients than those from healthy individuals (Fig. 7p, q), highlighting that a therapeutic drug, such as TRx0237, that targets/inhibits p-Tau Ser422, might prevent bone loss in general in addition to GIO in clinic.

## Discussion

**Fig. 8:**
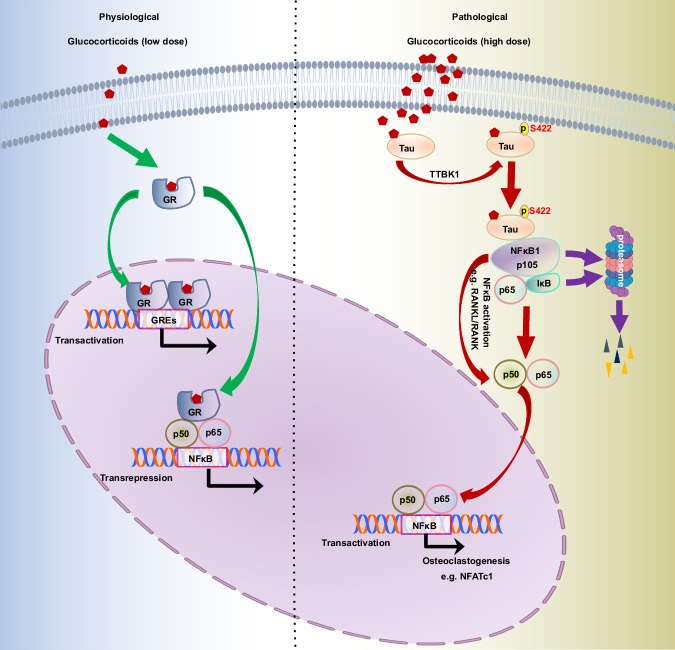
Model of mechanisms of dexamethasone-induced GIO. Upon binding to low-dose GC, GR is translocated into the nucleus and binds to the canonical GC response element or tethers with NF-κB to activate anti-inflammatory or repress pro-inflammatory gene expression, respectively. Alternatively, high-dose GC directly binds to Tau, and then recruits TTBK1 kinase to Tau, leading to the phosphorylation of Tau at Ser422. Activated p-Tau Ser422 facilitates the processing of NF-κB p105 into p50 and the nuclear translocation of NF-κB in RANKL-primed macrophages, resulting in enhanced osteoclastogenesis, and eventually causing GIO.

GC ligand stimulation results in the nuclear translocation of its canonical high-affinity receptor GR, where it 1) binds to GC response elements or 2) tethers itself to other DNA-bound transcription factors and alters transcription of downstream genes,^56^ leading to anti-inflammation and immunosuppression (Fig. 8). The presence of previously unrecognized GC receptors and the necessity to identify them have been actively discussed in the field. Starting from an unbiased systematic proteome-wide target identification strategy coupled with DARTS screen, followed by confirmation with solid phase binding and SPR assays, we identified Tau as a low-affinity receptor of GCs. Serial biochemical co-purification coupled with proteomic analyses led to the isolation of TTBK1 kinase and transcriptional factor p50 as two key downstream mediators of GCs/Tau signaling to mediate GC-dependent osteoclastogenesis and bone resorption (Fig. 8).

Although Tau is well known as a microtubule-binding protein and its aggregation is associated with various neurodegenerative diseases including Alzheimer's  disease and frontotemporal dementia,^57,58^ it has many faces and can also function as a signaling molecule.^39,59^ Here we report that upon binding with high-dose dexamethasone, non-microtubule-associated cytoplasmic Tau acts as a signaling molecule to tether TTBK1 kinase, leading to the phosphorylation of Tau at residue Ser422, followed by the processing of NF-κB p105 into p50 and its nuclear translocation, thereby, enhancing RANKL-primed osteoclastogenesis. Our findings that Tau is a low-affinity receptor of GCs and plays a vital role in GC-induced bone resorption, also help address the long lasting debates/questions concerning the molecular events behind GCs-induced osteoporosis.^37^

GIO is characterized by impaired bone remodeling, an active and dynamic process that controls the balance between bone resorption by osteoclasts and bone formation by osteoblasts. It is generally accepted that enhanced bone resorption results from the direct actions of GCs on osteoclasts and their secondary effects on osteoclasts-mediated regulation of osteoblasts, osteocytes and bone marrow adipocytes.^9^ In human patients, GIO manifests a rapid phase characterized by a decrease in bone mineral density (BMD), presumably due to enhanced bone resorption, followed by a gradual slower phase during which BMD decrease progressively due to compromised bone formation.^60^ In vitro and in vivo studies demonstrated that GCs directly enhance bone resorption/osteoclast activity.^61–66^ Although it was reported that GCs acted directly on osteoclasts to reduce bone density in vivo using TRAP-Hsd11b2 transgenic mice in which GCs was inactivated specifically in osteoclasts,^63^ the receptor which mediates this process has been controversial. A study reported that dexamethasone’s impact on osteoclast differentiation depends on GR by using osteoclast-specific *GR* deletion mice.^67^ In contrast, other investigators conducted similar studies using the same mouse model but were unable to demonstrate any osteoclastic phenotype.^35^ These contrasting findings imply the possible existence of receptors other than GR that mediate GCs’ regulation of osteoclast function. Our in vitro and in vivo data demonstrate that high-dose dexamethasone directly enhances osteoclast activity and bone resorption, depending on Tau, while it inhibits bone formation, depending on GR, which is in line with a previous report.^35^ Furthermore, our findings indicate that in the context of long-term high-dose dexamethasone treatment, the increased bone resorption has a more pronounced impact than the inhibition of bone formation. This is evident from the significant reduction in bone density observed in both wild-type and *GR* knockout mice, but not in *Tau* knockout mice.

Of note, we demonstrate that p-Tau Ser422 is a therapeutic target for treating GIO and identify TRx0237, previously reported to inhibit Tau aggregation,^68^ from an FDA-approved drug library as a drug which could be repurposed to block dexamethasone-induced bone loss. Combinatorial treatment with dexamethasone and TRx0237 enabled the beneficial anti-inflammatory action while averting the adverse bone resorption activity of dexamethasone in CIA, an inflammatory arthritis mouse model that resembles human RA. Also, we found p-Tau Ser422 was significantly elevated in patients with osteoporosis, suggesting that clinical inhibition of Tau phosphorylation at Ser422 might prevent bone loss in general in addition to GIO. TRx0237, a drug entering clinical phase III trial with known pharmacological and toxicological profiles, may represent a promising therapy for various GCs-associated adverse effects, particularly GIO, p-Tau Ser422-associated tauopathy and other pathological conditions, such as acute stress, in which GCs are markedly stimulated.^69^

The DARTS assay with live cells demonstrated that 10 µM high-dose dexamethasone was needed to obtain the clear protection of Tau from protease-mediated degradation, whereas protection of GR degradation could be seen from a dose as low as 10 nM dexamethasone. Moreover, 10 µM dexamethasone was required to enhance Tau-dependent downstream signaling and resultant osteoclast activity. The use of 10 mg/kg body weight of dexamethasone in mouse models is consistent with established protocols for inducing GIO, providing a robust model to study the underlying mechanisms and potential therapeutic interventions. Thus, the doses of 10 μM dexamethasone in vitro and 10 mg/kg body weight in our mouse models were chosen to elucidate the mechanistic pathways involved. The higher doses employed in our study serve to model extreme conditions of GC exposure, which can provide insights into the potential effects of and mechanisms underlying high-dose GC therapy, such as in severe inflammatory diseases or in certain cancer treatments. Nevertheless, we recognize the need to bridge these findings to clinically relevant scenarios. In future studies, we aim to investigate the GC/Tau pathway activation at lower, more therapeutically relevant dose range in vivo to better understand the clinical implications.

While our current study primarily focuses on the role of the Tau–GCs interaction in bone resorption and the efficacy of TRx0237 in preventing dexamethasone-induced bone loss, we recognize that GCs are associated with a variety of side effects, including insulin resistance, high blood glucose, disorders of lipid metabolism, myopathy, and immunosuppression. As a microtubule-binding protein, the presence of Tau in various cell types suggests that Tau may play a role in mediating GC effects beyond the skeletal system, and TRx0237, by inhibiting the Tau-GCs pathway, could potentially reduce GC-induced other side effects. Future studies will involve comprehensive in vitro and vivo experiments to evaluate the effects of GC–Tau interaction and TRx0237 on a range of GC-induced side effects.

Aggregated Tau protein is associated with a range of neurological disorders, including Alzermer’s disease. In Alzheimer’s disease, tau protein becomes hyperphosphorylated and forms insoluble filaments that accumulate into neurofibrillary tangles that lead to memory loss and other symptoms with p-Tau Ser422 serving as an aggregation marker for this process.^70^ Moreover, long-term GC therapy is associated with significant side effects, including neurodegenerative risks.^71^ There is evidence suggesting stronger female Alzheimer’s disease association with osteoporosis.^72^ In our study, we identify Tau pS422 as a critical phosphorylation site in GCs/Tau signaling to mediate GCs-induced osteoclast activity. Given the role of Tau in both bone and brain, our findings raise the possibility that high-dose GCs might similarly impact Tau phosphorylation and aggregation in neurons, potentially contributing to neurodegenerative disease progression. Further work can be pursued to disentangle the links between GCs-induced osteoporosis and neurodegenerative mechanisms, which may help in developing therapeutic strategies for these conditions.

In this study, we primarily utilized biochemical, biophysical, proteomic and genetic assays to pinpoint Tau as a low-affinity receptor for GCs. We then validated its functionality by employing various in vitro cell-based assays and in vivo genetically modified mice in both GIO and CIA models. Future structural approaches, such as cryo-EM and X-ray crystallography, are expected to provide additional insights into the interaction between GCs and Tau. We presented comprehensive in vitro and in vivo evidence demonstrating that Tau plays a critical role in high-dose GCs-induced osteoclastogenesis and bone loss. However, we acknowledge that utilizing osteoclast-specific *Tau* knockout mice would provide additional evidence to further assess the contribution of Tau in high-dose GCs-induced osteoclastogenesis and GIO. Unfortunately, the technical challenge arises from the unavailability of conditional *Tau* knockout mice. Additionally, using bone marrow chimeras with WT mice transplanted with tau-deficient bone marrow cells can limit tau deficiency to hematopoietic cells, including osteoclasts. This approach may aid in clarifying the cell-autonomous role of Tau in these cells in GIO model, warranting further exploration. The mechanisms that elucidate how a very small percentage of Tau dissociated from microtubules in response to high-dose dexamethasone can effectively mediate high-dose dexamethasone-induced osteoclastogenesis and bone loss merit further investigation. Additionally, a comprehensive exploration of the mechanisms underlying how Tau deficiency confers protection against inflammation in CIA is needed, although we recently reported the involvement of Tau-regulated macrophage polarization in this phenomenon.^73^

In summary, this study identifies Tau as a GCs-binding receptor with low affinity, thus providing a foundation for future discoveries relating to GCs/Tau pathway. Additionally, it also confirms that this pathway plays a vital role in GIO, one of the several well-recognized substantial side effects of GCs. While this study focuses on GIO, the discovery with GCs/Tau pathway may contribute to the understanding of diverse manifestations associated with high-dose GCs, and the results of this study may have a broader application to various GCs-associated side effects. Additionally, this pathway may be transiently activated under some stress conditions during which GC levels are well known to be elevated temporarily. Furthermore, this study may also contribute to the understanding of GCs activities in various Tau-related diseases and conditions. More importantly, we have successfully isolated TRx0237 as a drug that, through inhibiting GCs/Tau signaling, could effectively overcome dexamethasone-induced bone loss. It is expected that TRx0237 may also be therapeutic against other side effects of GCs in various diseases and conditions, including tauopathy. With the consideration that both GCs and Tau are involved in a plethora of pathophysiological and disease processes, the identification of Tau as a low-affinity receptor of GCs and the manipulation of this new Tau/GCs pathway not only enhance our understanding of GCs and Tau actions, but may also provide therapeutics for various GCs- and Tau-associated pathologies and conditions.

## Materials and Methods

### Mice

C57BL/6 (000664), *Tau*^*−/−*^ (007251),^30^ *GR*^*f/f*^ (021021)^28^ and *Rosa26a-CreERT2* (008463)^29^ mice were obtained from The Jackson Laboratory. *GR*^*f/f*^ mice were crossed with *Rosa26a-CreERT2* mice to generate inducible *GR* knockout mice. For activation of CreERT2 in adult mice, 150 mg/kg body weight of tamoxifen (Sigma-Aldrich) in sunflower seed oil (Sigma-Aldrich) was intraperitoneally injected into 10-week-old mice once a day for 5 consecutive days. Knockout efficiencies were analyzed 1 week after the last injection by qRT-PCR. *Tau*^*−/−*^ and WT mice were littermates generated from heterozygous interbreeding. All genetic models were on the C57BL/6 background, and sex- and age-matched littermates were used for experiments, typically at 3 months of age. All animals were housed on a 12-h light-dark cycle with ad libitum access to food and water in a specific pathogen-free environment. Animal protocols were approved by Institutional Animal Care and Use Committee (IACUC) of New York University Grossman School of Medicine (#160605).

### Identification of dexamethasone binding proteins using human proteome microarray

HuProt human proteome microarray version 4.0 (HuProt^TM^, CDI Laboratories), which is composed of ~ 20,000 human FL proteins with N-terminal glutathione S-transferase tag was used to isolate dexamethasone-binding proteins. Briefly, after blocking with 20 mM Tris-Cl, pH 7.5, 150 mM NaCl, 0.1% Tween-20, 5% BSA on an orbital shaker at room temperature for ≥ 2 h, the protein chips were incubated with 0.2 and 400 µM biotin-labeled dexamethasone, respectively, overnight at 4 °C. The next day, the protein chips were rinsed three times with phosphate-buffered saline (PBST) (with 0.1% Tween-20), followed by incubation with Cy5-conjugated streptavidin (1:1000, Invitrogen, Camarillo, CA, USA) in the dark at room temperature for 1.5 h. After washing three times with 1× PBST and then three times with Milli-Q water, the protein chips were centrifuged for 5 min in a 50 mL centrifuge tube. Finally, the protein chips were scanned with an Axon GenePix 4000B Microarray Scanner (Molecular Devices), and the probe signals were acquired using GenePix Pro6.0 software (Molecular Devices). The probes were considered detectable when the *z*-scores for both duplicates were greater than 2.8.

### Plasmids

The cDNA encoding FL Tau was amplified with specific primers 5’-atcgggatccgctgagccccgccaggagttc-3’containing *Hind*III restriction site and 5’-attatgtcgaccaaaccctgcttggccaggga-3’ containing *Sal*I restriction site using *Tau* cDNA (Origene) as a template. The digested sequence was cloned into pCMV-tag2b vector (Agilent). Expression plasmids for serial FLAG-tagged deletion mutants of tau were also constructed in the pCMV-tag2b vector. The DNA sequences were confirmed by DNA sequencing performed by Psomagen.

### Site-directed mutagenesis

Eight residues at Asp193, Ser202, Thr205, Thr212, Lys225, Ser238, Ser396, and Ser422 of Tau were individually mutated using Site-Directed Mutagenesis Kit (Agilent, #200522) in accordance with the manufacturer’s protocol. Specifically, Asp at residue 193 was mutated to Glu, Ser at residue 202 was mutated to Phe, Thr at residue 205 was mutated to Ile, Thr at residue at 212 was mutated to Asn, Lys at residue 225 was mutated to Asn, Ser at residue 238 was mutated to Tyr, Ser at residue 396 was mutated to Pro, and Ser at residue 422 was mutated to Pro, respectively.

### DARTS assay

DARTs assay was performed as previously described.^74^ For determining the molecular targets which were protected from proteinase-mediated degradation, intact Raw264.7 cells were treated with 10 µM dexamethasone, lysates were extracted with M-PER™ Mammalian Protein Extraction Reagent (Invitrogen) and subjected to protease (Sigma) digestion for 15 min at room temperature. Protease inhibitor cocktail was added to lysates followed by a 10 min incubation on ice to stop the digestion. The mixtures were separated on SDS-PAGE gel, followed by Coomassie staining.

For determining the dose-dependent curve for Tau and GR, Raw264.7 cells were lysed with M-PER™ Mammalian Protein Extraction Reagent, and incubated with serial doses of dexamethasone for 1 h with gentle rotation at room temperature. Then the mixtures were digested with pronase and subjected to western blot analysis with anti-Tau and anti-GR antibodies.

For determining the amino acid critical for dexamethasone binding, HEK293T cells were transfected with FL Tau, serial deletion Tau mutants or site-mutated Tau expression plasmids using lipofectamine 2000 according to the manufacturer’s protocol. 48 h after transfection, lysate was extracted by M-PER™ Mammalian Protein Extraction Reagent and mixed with 10 µM dexamethasone for 1 h with rotation at room temperature. Pronase (Sigma) at different concentrations was added in the reaction for 15 min at room temperature. Protease inhibitor cocktail was added to stop the digestion by incubation with the mixture on ice for 10 min. Samples were boiled in SDS loading buffer for immunoblot detection with anti-FLAG antibody.

### Solid phase binding

To compare the binding affinity between dexamethasone and Tau in vitro, microtiter plates were coated with 500 ng of Tau (SAE0076, Sigma-Aldrich) in 100 μL of Tris-buffered saline (TBS) buffer (50 mM Tris-HCl, 150 mM NaCl, pH 7.4) overnight at 4 °C. Wells were blocked using 1% bovine serum albumin (BSA) in TBS buffer for 2 h at 37 °C. After washing with TBS and 0.05% Tween, various amounts of biotin-labeled dexamethasone (sc-499756; Santa Cruz) were added. Bound protein from the liquid phase was detected using streptavidin-HRP (1:500, SA10001, Invitrogen) for 30 min at room temperature. The plates were washed with Tris-buffered saline with Tween 20 (TBST) 6 times and the reaction was visualized following a 15 min incubation with substrate 3,3’,5,5’-Tetramethylbenzidine (002023, Invitrogen). Then the signal development was stopped by H_2_SO4. The absorbance at 450 nm was measured by SpectraMax i3X microplate reader (Molecular Devices, LLC., CA, USA).

### Molecular docking simulations

Maestro v11.1 (Schrodinger LLC, MA, USA) was used for performing molecular docking simulations. The structures of prednisolone and dexamethasone were downloaded from PubChem database. The energy of the ligand structures were minimized in Macromodel v11.5 module using OPLS3 force field, followed by a ligand preparation process in LigPrep v4.1 module according to the following parameters to generate low-energy 3D structure: different protonation states at pH 7.0 ± 2.0, and all possible tautomers and ring conformations. The prepared ligand was subjected to dock into the Tau protein model. The Tau protein structure (PDBDEV_ 00000033)^75^ was prepared using the Protein Preparation Wizard implemented in Maestro v11.5. The docking grid (length: 35 Å) was generated by selecting residues 150–244 as centroid. Flexible docking was performed by Glide v7.4 extra precision (XP) method. To further get an optimal simulation of binding, the best-docked pose from Glide XP docking was subjected to induced-fit docking. The docked conformations of the tested ligand were ranked based on docking score, glide energy, as well as by analyzing the binding site interactions, and the best docked pose of ligand–protein complex was selected for graphical analysis.

### SPR analysis

To further determine the binding affinity between GCs and Tau, SPR analysis was performed by Essai Sciences LLC (Stillwater, OK). All experiments were carried out on the SensiQ Pioneer FE SPR platform. The buffer used was 10 mM HEPES, pH 7.4, 150 mM NaCl, and .01% Tween-20. Channels 1, 2, and 3 were activated by injection of a solution of 200 mM EDC and 50 mM NHS for six minutes. Tau, in 10 mM sodium acetate, pH 5.5, was then injected over channels 1 and 2, with channel 3 left activated, but empty, as a reference. All three channels were then injected with 1 M ethanolamine, pH 8.0, for 2 min. Small molecule analytes were dissolved in DMSO at a concentration of 10 mM. Analytes were further diluted in buffer to concentrations of 200 μM and 40 μM, at 4% DMSO. Experimental assays used the OneStep dynamic injection method which employs an analyte concentration gradient generated during injection using an analyte dispersion method. This method is particularly useful for small molecule analysis where possible molecular affinity toward immobilized target is unknown, and allows for less analyte consumption and faster assay results which are comparable to fixed concentration injection series.

### Generation of *Tau*, *GR* knockout cell lines using CRISPR-Cas9

To generate *Tau* or *GR* knockout Raw264.7 cells, target sgRNA oligonucleotides were annealed and cloned into *BSM*B1 restriction sites of a lentiCRISPRv2 vector (52961, Addgene). The sequence for each sgRNA is as follows: 5’-GCGGAGACACCCCGAACCAGG-3’ and 5’-GTGGAGCGGAGGAACCAGGGT-3’ for mouse *Tau*; 5’-GTGGACTTGTATAAAACCCTG-3’ and 5’-GTGGTACATCTGTCCTCCAG-3’ for mouse *GR*. Co-transfection of CRISPR plasmid, psPAX2 (12260, Addgene), and pMD2.G (12259, Addgene) into HEK293T (ATCC) was performed with Lipofectamine 2000 to produce the lentivirus. Then Raw264.7 cells were infected with the collected lentivirus for 18 h, followed by selection with 2 μg/mL puromycin (Gibco) for 2 days. Cells were expanded and evaluated for knockout efficiency by western blot. Two or three distinct clones for *Tau* or *GR* knockout cell lines were established, and the results from representative clone were presented.

To generate *Tau* or *GR* knockout THP-1 cells, target sgRNA oligonucleotides were annealed and cloned into *BSM*B1 restriction sites of a lentiCRISPRv2-mCherry vector (99154, Addgene) and a lentiCRISPRv2-GFP vector (82416, Addgene). The sequence for each sgRNA is as follows: 5’-GTGCTAAGAGCACTCCAACAG-3’ and 5’-GAAACGAAGATCGCCACACCG-3’ for human *Tau*; 5’-GTGGTACATCTGTCCTCCAG-3’ and 5’-GCTTTAAGTCTGTTTCCCCCG-3’ for human *GR*. For electroporation, the entire supplement is mixed with the Nucleofector Solution from Cell Line Nucleofector™ Kit V (VCA-1003, Lonza), and 2 µg plasmid DNA is added to 100 µL room temperature Nucleofector Solution. 2 × 10^6^ THP-1 cells were collected by centrifugation with resuspension of the cell pellet carried out in 100 µL room temperature Nucleofector Solution and DNA mixture. The cell/DNA suspension was transferred into the cuvette and applied with the Nucleofector Program X-001 using NucleofectorTM System.^76^ The transfected cells were cultured for 48 h prior to sorting the successful transfectants according to mCherry signal and the cells were prepared for single-cell cloning through fluorescence-activated cell sorting. Clones were expanded and evaluated for knockout efficiency by western blot. Two or three distinct clones for *Tau*, *GR*, or *Tau* and *GR* double knockout cell lines were established, and the results from one representative one clone was presented.

### IP and immunoblot analysis

For IP endogenous protein, 1 × 10^8^ Raw264.7 cells treated with 10 µM dexamethasone for 30 min were collected and lysed with 750 µL ice-cold lysis buffer (phosphate-buffered saline, pH 7.4, 1.5% (v/w) Triton X-100, 1 mM Na_3_VO_4_, 1 mM NaF, 1 mM DTT, with protease inhibitor cocktail) on ice for 15 min, followed by sonication and centrifugation at 4 °C for 15 min. For IP TTBK1, 2 µg anti-Tau antibody (MA5-12808, Invitrogen) or mouse IgG (sc-3795, Santa Cruz) was bound to 45 µL protein A/G agarose (sc-2003, Santa Cruz) and then added to the cleared cell lysate and incubated with rotation overnight at 4 °C. For IP p105/p50, 2 µg anti-tau antibody or mouse IgG was immobilized onto magnetic beads using Pierce™ Crosslink Magnetic IP Kit (88805, ThermoFisher Scientific) and then added to the cleared cell lysate and incubated with rotation overnight at 4 °C. Immunoprecipitates were washed three times with lysis buffer and mixed with an equal volume of 2× SDS sample buffer, followed by immunoblot analysis.

For IP of ectopically expressed FL and mutated Tau, 1 × 10^8^ Raw264.7 cells were transfected with different expression plasmids. At 48 h after transfection, cells were treated with 10 µM dexamethasone for 30 min and lysed with 750 µL ice-cold lysis buffer on ice for 15 min, followed by sonication and centrifugation at 4 °C for 15 min. Anti-FLAG M2 affinity Gel (A2220, Sigma) was added into the cleared cell lysate and incubated with rotation overnight at 4 °C. The beads were washed with lysis buffer 5 times at 4 °C and then mixed with an equal volume of 2× SDS sample buffer, followed by immunoblot analysis of TTBK1 and p105/p50.

For immunoblot analysis, the protein samples were resolved on 8% or 10% resolving gel and transferred to 0.45-µm nitrocellulose membrane (1620097, Bio-Rad) using a wet transfer system. Proteins were detected by incubation with 1:1000 dilutions of primary antibodies, washed and incubated with appropriate secondary antibodies, and detected after incubation with Western Lightning™ Plus Chemiluminescence Reagent (NEL104001, ThermoFisher Scientific) with ChemiDoc Imaging System (17001401, Bio-Rad). The following antibodies were used for immunoblotting: anti-Tau (MA5-12808, Invitrogen), anti-GR (MAI-510, ThermoFisher Scientific), anti-TTBK1 (PA5-20686, ThermoFisher Scientific), anti-p105/p50 (12540 S, Cell Signaling technology), anti-Tau pSer202/Thr205 (MN1020, ThermoFisher Scientific), anti-Tau pSer396 (44-752 G, ThermoFisher Scientific), anti-Tau Ser422 (44-764 G, ThermoFisher Scientific), anti-GAPDH (60004-I-Ig, Proteintech), anti-FLAG (F7425, Sigma), anti-CTCF (2899 S, Cell Signaling Technology), horseradish peroxidase conjugated anti-mouse IgG (115-035-003, Jackson ImmunoResearch Laboratories), and horseradish peroxidase conjugated anti-mouse IgG (111-035-003, Jackson ImmunoResearch Laboratories). Quantification of bands was performed using ImageJ software.

### Biochemical co-purification and mass spectrometry

To isolate the kinases responsible for phosphorylating Tau, control and *Tau* knockout Raw264.7 cells were treated with or without 10 µM biotin labelled dexamethasone for 30 min, and lyzed with lysis buffer (20 mM potassium phosphate buffer, 0.15 M NaCl, pH 7.5, with proteinase inhibitor cocktail). After centrifugation, the supernatant was incubated with Streptavidin Magnetic Particles (11641778001, Sigma) for 2 h at 4 °C. The magnetic particles were then washed with lysis buffer 5 times, followed by elution with 0.1 M glycine-HCl, pH 2.5. These samples were then analyzed by mass spectrometry, performed by NYU Proteomics Laboratory. All MS/MS spectra were collected using the following instrument parameters: resolution of 15,000, AGC target of 5e4, maximum ion time of 120 ms, 1 microscan, 2 m/z isolation window, fixed first mass of 150 m/z, and NCE of 27. MS/MS spectra were searched against a Uniprot Human database using Sequest within Proteome Discoverer 1.4.

To isolate the transcription factors downstream of dexamethasone-bound Tau, Raw264.7 cells were transfected with FLAG or FLAG-tagged Tau. Forty-eight hours post transfection, cells were treated with 10 µM dexamethasone for 30 min. FLAG or FLAG-tagged Tau was affinity-purified on FLAG M2 affinity gel, and the mixture was eluted with FLAG peptide.

The eluted samples were then analyzed by mass spectrometry, performed by NYU Proteomics Laboratory. All MS/MS spectra were collected using the following instrument parameters: resolution of 15,000, AGC target of 5e4, maximum ion time of 120 ms, 1 microscan, 2 m/z isolation window, fixed first mass of 150 m/z, and NCE of 27. MS/MS spectra were searched against a Uniprot Human database using Sequest within Proteome Discoverer 1.4.

### Cell culture and treatment

The human monocytic THP-1 cells, murine macrophage RAW264.7 cells, and HEK293T cells were obtained from ATCC. RAW264.7 cells and HEK293T cells were maintained in DMEM with 10% FBS and 1% antibiotics (1% penicillin-streptomycin (15140-122, ThermoFisher Scientific)) at 37 °C under 5% CO_2_ in a humidified incubator. THP-1 cells were maintained in RPMI-1640 with 10% FBS and 1% penicillin-streptomycin in a humidified atmosphere of 5% CO_2_ in a humidified incubator.

To determine the effect of dexamethasone on phosphorylation status of Tau, RAW264.7 cells were serum-starved overnight in DMEM medium, then stimulated with dexamethasone for the indicated time. To determine which kinase is responsible for phosphorylating Tau Ser422, RAW264.7 cells were pre-treated with 10 nM serine/threonine protein kinase TAO1 inhibitor (TAO kinase inhibitor) (HY-112136, MedChemExpress), 10 nM serine/threonine protein kinase Nek9 inhibitor (Dabrafenib) (HY-14660, MedChemExpress), 5 nM leucine-rich repeat serine/threonine protein kinase 2 inhibitor (MLi-2) (HY-100411, MedChemExpress), 10 mM GSK-3β inhibitor (LiCl) (7447-41-8, ThermoFisher Scientific) or 10 μM p50 inhibitor (Andrographolide) (HY-N0191, MedChemExpress) for 1 h prior to treatment with 10 µM dexamethasone for 30 min for western blot analysis or differentiation into osteoclast with 50 ng/mL RANKL and 10 µM dexamethasone for 5 days.

To knock down TTBK1 and CTCF in Raw264.7 cells, cells were transfected with TTBK1 siRNA (sc-154747, Santa Cruz), CTCF siRNA (SR419676, Origene) or scramble negative control siRNA (SR30004, Origene) using Lipofectamine 2000 (11668019, Invitrogen) for 48 h. After transfection, the knockdown efficiency was determined by western blot.

### Immunofluorescence staining and confocal microscopy

For the immunofluorescence staining of total Tau, phospho-Tau Ser422, and tubulin, Raw264.7 cells plated in the 8-well chambers (Z734853, Sigma) were treated with PBS, 100 ng/mL RANKL (390-TN/CF, R&D Systems), and 100 ng/mL RANKL plus 10 µM dexamethasone for 15 min. The cells were then fixed with 100% methanol (for total Tau) or 100% acetone (for phospho-Tau Ser422), for 10 min at room temperature, followed by permeabilization in 0.1% Triton X-100 for 10 min before blocking in 1% BSA for 1 h at room temperature. The following primary antibodies were incubated with cells at 4 °C overnight: Tau antibody (1:100, MA5-12808, Invitrogen); phospho-Tau Ser422 antibody conjugated with Alexa Fluor® 647 (1:100, ITA0917, G-Biosciences). For total Tau, the cells were incubated with Alexa Fluor® 647 anti-mouse IgG (1:200, A-21235, Invitrogen) for 1 h at room temperature in the dark. Next, cells were incubated with anti-α-Tubulin antibody conjugated with Alexa Fluor® 488 (1:200, 16-232, Millipore Sigma) for 2 h at room temperature. DAPI (1:1000, D1306, ThermoFisher Scientific) was used to stain the nuclei for 10 min at room temperature.

For the immunofluorescence staining of p105/50, RAW264.7 cells grown on 8-well chambers were treated with PBS, 100 ng/mL RANKL, or 100 ng/mLRANKL plus 10 µM dexamethasone for 4 h. After treatment, the cells were fixed with 4% paraformaldehyde (PFA) for 10 min at room temperature, then permeabilized in 0.1% Triton X-100 for 10 min, followed by blocked in 1% BSA for 1 h at room temperature. Cells were then incubated with anti-NF-κB p105/p50 antibody (1:100, 13586 S, Cell Signaling Technology) at 4 °C overnight, followed by anti-Rabbit IgG conjugated with Alexa Fluor® 488 (1:200, A-11008, Invitrogen) for 1 h at room temperature. DAPI (1:1000) was used to stain the nuclei for 10 min at room temperature.

For the immunofluorescence staining of total Tau and TTBK1, Raw264.7 cells plated in the 8-well chambers were treated with or without 10 µM dexamethasone for 30 min. Cells were fixed with 4% paraformaldehyde in PBS pH 7.4 for 10 min at room temperature, then permeabilized using 0.1% TritonX-100 for 10 min at room temperature. Cells were blocked by incubating with 5% BSA for 1 h at room temperature. Immunofluorescence staining was performed by using anti-Tau antibody (1:100, MA5-12808, ThermoFisher Scientific) and anti-TTBK1 antibody (1:100ABN348, Millipore). These primary antibodies were detected with Alexa Fluor 488-labeled goat anti-mouse IgG (1:200, A-11001, ThermoFisher Scientific) and Alexa Fluor 546-labelled goat anti-rabbit IgG (1:200, A-10040 ThermoFisher Scientific). DAPI (1:1000) was used to stain the nuclei for 10 min at room temperature. All images were taken on a Zeiss laser scanning microscope (LSM) 780 with a 63×, 1.4 NA oil objective. Fluorescence intensity was quantified using ImageJ software.

### Osteoblastogenesis and osteoclastogenesis

The bone marrow cells isolated from 12-week-old male WT, *Tau*^*−/−*^, and *GR*^*−/−*^ mice were cultured in MEM-α with 10% FBS and 1% penicillin-streptomycin (15140-122, ThermoFisher Scientific) for at least 24 h. For osteoblastogenesis, the adherent cells were differentiated in osteogenic induction medium (10% FBS, 100 µg/mL ascorbic acid and 10 mM β-glycerophosphate in MEM-α) in the absence or presence of 10 nM or 10 µM dexamethasone. For osteoclastogenesis, the non-adherent cells were cultured with 20 ng/mL M-CSF (576406, Biolegend) for 3 days, and switched to the differentiation medium including 20 ng/mL M-CSF, 50 ng/mL RANKL (390-TN-010/CF, R&D Systems), with or without 10 nM or 10 µM dexamethasone. To differentiate Raw264.7 cells to osteoclasts, cells were treated with 50 ng/ml RANKL in the presence or absence of 10 µM dexamethasone. To differentiate THP-1 cells to osteoclasts,^77^ the cells were incubated with 100 ng/mL phorbol‑12 myristate‑13 acetate for 48 h to differentiate into adherent macrophages, and subsequently, 50 ng/mL RANKL and 20 ng/mL M-CSF in the presence and absence of 10 µM dexamethasone were added into the adherent cells. The medium was replaced every day.

After differentiation into osteoblasts for 10 days and osteoclasts for 5 days, respectively, cell apoptosis was detected using In Situ Cell Death Detection Kit (11684795910, Sigma) in accordance with the manufacturer’s instructions. Briefly, the cells were fixed with a freshly prepared fixation solution (4% paraformaldehyde in PBS, pH 7.4) for 1 h at room temperature. After rinsing with PBS, the cells were then incubated with permeabilization solution (0.1% Triton X-100 in 0.1% sodium citrate) for 2 min on ice. After washing twice with PBS, 50 μL of TUNEL reaction mixture was added. Then the cells were incubated in a humidified atmosphere for 1 h at 37 °C in the dark. DAPI (1:1000) was used to stain the nuclei for 10 min at room temperature. The samples were then analyzed under the confocal microscope.

After differentiation into osteoblasts for 21 days from primary bone marrow cells, cells were fixed in 4% PFA and stained with Alizarin Red S (A5533-25G, Sigma-Aldrich). At the end of differentiation into osteoclasts from bone marrow cells for 7 days, Raw264.7 cells for 5 days or THP-1 cells for 14 days, cells were stained for osteoclasts using the TRAP kit (387 A, Sigma-Aldrich), the number of TRAP-positive multinucleated cells containing more than 3 nuclei and the number of nuclei per osteoclast were counted using microscopy.

Bone resorption activity was measured using a fluoresceinated calcium phosphate-coated OsteoAssay plates. The coated calcium phosphate is first bound to fluoresceinamine-labeled chondroitin sulfate, which is released from the calcium phosphate layer into conditioned medium by osteoclastic resorption activity. To reveal osteoclast resorption pits, bone marrow cells grown on OsteoAssay plate (CSR-BRA-48, Cosmo Bio Co., Ltd) were stimulated with 20 ng/mL M-CSF, 100 ng/mL RANKL with or without 10 µM dexamethasone for 7 days. At the end of stimulation, the conditioned medium with fluoresceinamine-labeled chondroitin sulfate from each well was transferred to 96-well black plate and mixed with bone resorption assay buffer in accordance with the manufacture’s guidelines. The fluorescence intensity of each well was detected with an excitation wavelength of 485 nm and an emission wavelength of 535 nm. Cells were then removed by sodium hypochlorite (5%) for 5 min. The bottom of the well was photographed by a light microscope, and the resorption area per well was calculated by ImageJ software.

### Primary osteocyte isolation

Osteocytes were isolated from mouse long bones using a method described by ref.^78^ Briefly, tibiae and femurs were aseptically dissected, and bone marrow was removed. The bones from 3 different mice with the same genetic background were pooled together and subjected to nine sequential digestions to remove periosteum, fibroblast, osteoblast, osteoclast and other adherent cells. Collagenase solution (0.25 mg/mL collagenase type I and 0.75 mg/mL collagenase type II) was prepared in αMEM, and EDTA solution (5 mM) was prepared in magnesium and calcium-free PBS with 1% BSA.

### Quantitative RT-PCR

Total RNA was isolated from cultured cells or pulverized tibia using RNeasy Mini Kit (74106, Qiagen). cDNA was prepared using 1 µg RNA with the High-Capacity cDNA Reverse Transcription Kit (4368813, Applied Biosystems). SYBR green-based (4367659, Applied Biosystems) quantitative PCR was performed in triplicate on the Real-Time PCR System (Applied Biosystems) using the following primers: *NFATc1* forward 5’- CATCCTGTCCAACACCAAAGTC-3’ and reverse 5’-GTGTTCTTCCTCCCGATGTCTG; *CTSK* forward 5’-ATGGAAGAAGACTCACCAGAAG-3’ and reverse 5’-CCACTTCTTCACTGGTCATGTC-3’; *CTR* forward 5’- GTCTTGCAACTACTTCTGGATGC-3’ and reverse 5’-CGTGGATAATGGTTGGCACTATC-3’; *TRAP* forward 5’- CAGCAGCCAAGGAGGACTAC-3’ and reverse 5’-CACATAGCCCACACCGTTCTC-3’; *OPG* forward 5’-TTGCCTGGGACCAAAGTGAATG-3’ and reverse 5’-GCTGCTTTCACAGAGGTCAATG; *Runx2* forward 5’-ATGCTTCATTCGCCTCACAAA and reverse 5’-ATGCTTCATTCGCCTCACAAA; *OCN* forward 5’-CTGACCTCACAGATCCCAAGC and reverse 5’-TGGTCTGATAGCTCGTCACAAG; *Col1a* forward 5’-TGAACGTGGTGTACAAGGTC-3’ and reverse 5’-CCATCTTTACCAGGAGAACCAT; *BSP* forward 5’-AAGCACAGACTTTTGAGTTAGC-3’ and reverse 5’-ACTTCTGCTTCTTCGTTCTCAT; *Gapdh* forward 5’-CCCAGAACATCATCCCTGCATC-3’ and reverse 5’-TCTTGATGTCATCATACTTGGCAG-3’. mRNA levels were normalized to *Gapdh* and reported as relative mRNA fold change.

### ELISA analysis

ELISA-based assay was performed to detect the inhibitory effects of FDA-approved drugs on dexamethasone-dependent p-Tau Ser422. Briefly, ELISA plates were coated with 12.5 ng anti-Tau antibody (MA5-12808, Invitrogen) diluted in PBS overnight at 4 °C and blocked with 5% BSA in PBS for 2 h at room temperature. THP-1 cells were serum-starved overnight in RMPI-1640 medium, then pre-treated with or without individual drug in an FDA approved drug library (L1300, Selleckchem) for 1 h prior to stimulation with 10 µM dexamethasone for 30 min. Cells were lyzed with lysis buffer (50 mM HEPES, 10 mM MgCl_2_, 1% SDS, 150 mM NaCl with protease and phosphatase inhibitor cocktail), the subsequent supernatant was collected after centrifugation and protein concentration was determined using Pierce™ BCA Protein Assay Kit (23225, ThermoFisher Scientific). The standard curve was prepared by adding serial dilution of the supernatant collected from dexamethasone-treated THP-1 cells with a protein concentration of 0–400 ng/mL into the plates and the incubation with anti-Tau antibody coated-plates at room temperature for 2 h. Alternatively, for inhibition of dexamethasone-dependent activation of Tau phosphorylation at Ser422 by drugs, 200 ng/mL supernatant collected from individual library drug plus dexamethasone-treated cells was added into the plates and incubated for 2 h at room temperature. After washing, 12.5 µg/mL biotinylated phosphor-Tau antibody was then added into the plates and incubated for another 2 h at room temperature. After washing with PBST 6 times, the bound protein-antibody mixture was detected using streptavidin-HRP (1:500, SA10001, Invitrogen) for 30 min at room temperature. The plates were washed with PBST 6 times and the reaction was visualized by following 15 min incubation with substrate 3,3’,5,5’-Tetramethylbenzidine (002023, Invitrogen) with signal development stopped by H_2_SO_4_. The absorbance at 450 nm was measured by a microplate reader.

ELISA kits were used to detect levels of RANKL (MTR00, R&D Systems), OPG (MOP00, R&D Systems), CTX-1 (MBS9141384, MyBiosource), PINP (MBS2500076, MyBiosource) in sera collected from indicated murine models in accordance with the manufacturer’s instruction.

### ChIP-qPCR

Raw264.7 cells were treated with or without 50 ng/mL RANKL for 1 h, followed by fixation with 1% formaldehyde for 10 min at room temperature. ChIP was performed using antibodies against p50 and p65, following the manufacture instructions (ThermoFisher Scientific). The purified DNA was analyzed by PCR with primers specific to the *NFATc1* promoter: GGACGCCCATGCAATCTGTTAG (sense) and GTGCCCTGAGAAAGCTACTCTC (antisense).^79^ qPCR results from immunoprecipitated samples were normalized to that from input DNA.

### GIO model

Twelve-week-old male and female WT, *Tau*^*−/−*^ and *GR*^*−/−*^ mice received intraperitoneal injections of PBS or 10 mg/kg body weight dexamethasone (D2915, Millipore Sigma) daily for five consecutive weeks. Mice were then sacrificed, and long bones (tibia and femur), vertebrae (L1 and L2), and sera were collected for further study.

To determine the therapeutic effect of TRx0237 on dexamethasone-induced bone loss, twelve-week-old male WT, *Tau*^*−/−*^, and *GR*^*−/−*^ mice were intraperitoneally injected with PBS, 10 mg/kg body weight dexamethasone, or 10 mg/kg body weight dexamethasone plus 4 mg/kg body weight TRx0237 (S7762, Selleckchem) daily for five consecutive weeks. After treatment, all mice were sacrificed, and long bones (tibia and femur), vertebrae (L1 and L2), and sera were collected for further study.

### CIA induction and assessment

Twelve-week-old WT, *Tau*^*−/−*^, and *GR*^*−/−*^ male mice were immunized intradermally with 100 μL emulsion containing an equal volume of chicken type II collagen (20012, Chondrex) and complete Freund’s adjuvant (7001, Chondrex) at the base of the tail (day 0). Booster injections were performed with chicken type II collagen emulsified in incomplete Freund’s adjuvant (7002, Chondrex) on day 19. Seven days after booster injections (day 26), the mice with CIA phenotype were intraperitoneally injected with PBS or 10 mg/kg body weight dexamethasone daily for 5 consecutive weeks. To determine the therapeutic effect of TRx0237 on dexamethasone-induced bone loss in CIA mice, WT mice with CIA phenotype were given daily intraperitoneal injections of PBS, dexamethasone (10 mg/kg body weight), or dexamethasone (10 mg/kg body weight) plus TRx0237 (4 mg/kg body weight) for 5 consecutive weeks. Meanwhile, the clinical score was assessed individually every other day until euthanasia using the following system: 0 = no erythema and swelling; 1 = erythema and mild swelling confined to the tarsals or ankle joint; 2 = erythema and mild swelling extending from the ankle to the tarsals; 3 = erythema and moderate swelling extending from the ankle to metatarsal joints; 4 = erythema and severe swelling encompass the ankle, foot, digits, or ankylosis of the limb. Each paw was scored and scores of four paws were summed to give a total clinical score with a maximum possible score of 16 for each mouse. After five weeks of treatment, the mice were sacrificed (day 62), and long bones (tibia and femur), hind paws, and sera were harvested for further study.

### DEXA scan analysis

At the end of the establishment of the mouse model, mice were anesthetized using 200 µL anesthetic agent containing 20 mg/mL ketamine and 2 mg/mL xylazine, and the whole body was scanned on a Lunar Piximus bone densitometer (GE Medical System Lunar Corp.). The instrument was calibrated before each scanning session using a Phantom with known BMD according to the manufacturer’s instruction. The bone mineral density was analyzed using Lunar Piximus 2.10 software.

### µCT analysis

Long bones (tibia and femur) and vertebrae (L1 and L2) from WT, *Tau*^*−/−*^, and *GR*^*−/−*^ mice were fixed in 4% PFA for 24 h and then stored in 70% ethanol. The fixed bone samples were scanned using the high-resolution SkyScan microCT system (SkyScan 1172. Kontich, Belgium). The scanning method and settings are: the samples were scanned using a 10-MP digital detector, 10 W of energy (60 kV and 167 mA), and a pixel size was 9.7 microns, exposure 925 ms/frame rotation step 0.3 degrees with ×10 frame averaging, 0.5 mm Aluminum filter and scan rotation was 180 degrees. After scanning, the radiographs were reconstructed using NRecon software (version 1.7.3.0; Bruker microCT, Kontich, Belgium). Reconstruction was made with NRecon using GPU acceleration. Gaussian smoothing was applied with a 2 voxel radius, ring artefact and beam hardening corrections were applied in reconstruction. Ring artefact reduction was set to 7 pixels. Beam hardening correction was set to 40%. After reconstruction, the parameters of trabecular bone of tibia, femur, and vertebrae (L1 and L2) including BV/TV (%), Tb.Th (μm), and Tb.N (1/mm), were analyzed using CT Analyser (CTan) v.1.18.8.0 (Bruker). The 3D µCT images were obtained by CTvox v.3.3.1 software (Bruker). The region of interest was defined as follows: starting at a distance of 100 slices from the distal growth plate of the femur and extending a further distance of 200 slices in the proximal direction for the femur; starting at a distance of 50 slices from the proximal growth plate of the tibia and extending a further distance of 200 slices in the distal direction for tibia. The trabecular bone of vertebrae L1 and L2 was analyzed.

### Dynamic histomorphometric analysis

For calcein labeling of non-decalcified bone specimens, mice were intraperitoneally injected with 100 µL 2 mg/mL calcein (C0875, Sigma-Aldrich) in a 2% sodium bicarbonate solution per 20 g body weight at day 8 and day 3 before euthanasia. The femoral trabecular bone was analyzed in a 1.5 cm long region of interest starting 200 µm under the mineralized front of the growth plate.

### Histological analysis

Dissected, soft-tissue specimens were fixed in 4% PFA at 4 °C overnight, and then decalcified with 10% EDTA (pH 7.2) for 2 weeks for long bones and vertebrate, and 4 weeks for hind paws at 4 °C with a change of EDTA every other day. After samples were embedded in paraffin, 6-μm serial paraffin sections were prepared. Representative sections were stained with freshly prepared H&E and TRAP. H&E staining was performed according to the following order: two changes of xylene 2 min each; two changes of 100% ethanol 2 min each; one change of 95% ethanol for 2 min; one change of 80% ethanol for 2 min; one change of distilled water for 2 min; one change of hematoxylin solution (3801570, Leica Biosystems) for 2 min; one change of distilled water for 2 min; one change of differentiating solution (3803590, Leica Biosystems) for 2 min; one change of distilled water for 2 min; one change of blue buffer (3802915, Leica Biosystems) for 2 min; one change of distilled water for 2 min; one change of 95% ethanol for 2 min; one change of eosin solution (3801606, Leica Biosystems) for 2 min; one change of 95% ethanol for 2 min; two changes of 100% ethanol 2 min each; two changes of xylene 2 min each. The stained sections were mounted with Cytoseal XYL (83124, ThermoFisher Scientific). Inflammation scores were obtained based on the following criteria: 0 = no inflammation; 1 = slight thickening of the lining layer or some infiltrating cells in the underlying layer; 2 = slight thickening of the lining layer plus some infiltrating cells in the underlying layer; 3 = thickening of the lining layer, an influx of cells in the underlying layer, and presence of cells in the synovial space; 4 = synovium highly infiltrated with many inflammatory cells. Sections were stained for TRAP using the following protocol: two changes of xylene 2 min each; one change of 100% ethanol for 2 min; one change of 95% ethanol for 2 min; two changes of distilled water 2 min each. Then the sections were incubated with TRAP staining solution at 37 °C for 60 min and counterstained with 0.02% fast green for 2 min, followed by dehydrating through 95% ethanol, 100% ethanol, and xylene 5 s each. All images were captured using the Zeiss microscope (Axio Scope A.1, Carl Zeiss, LLC). The quantification of osteoclast surface and osteoblast surface on TRAP- and H&E-stained sections were performed with ImageJ software.

### Immunohistochemistry and immunofluorescence staining

For immunohistochemistry staining, deparaffinized and hydrated sections were incubated with 0.1% trypsin for antigen retrieval (30 min, 37 °C), followed by incubation with 3% H_2_O_2_ (30 min, 4 °C). After blocking with the solution containing 3% BSA and 20% goat serum for 60 min at 37 °C, sections were incubated with primary antibodies against phospho-Tau (Ser 422) (1:100, 44-764 G, Invitrogen) overnight at 4 °C. Then biotinylated anti-rabbit secondary antibodies (1:200, BA-1000-1.5, Vector Laboratories) were added into the sections for 60 min at room temperature. After washing with PBS, the Vectastain Elite ABC kit (PK-6100, Vector Laboratories) was used to amplify signals. Sections were then incubated with 0.5 mg/mL 3,3-diaminobenzidine in 50 mM Tris-Cl substrate (Sigma-Aldrich) until desired stain intensity developed, and counterstained with 1% methyl green.

For immunofluorescence staining, deparaffinized and hydrated sections were incubated with 0.1% trypsin for antigen retrieval (30 min, 37 °C). Subsequently, sections were blocked with 10% goat serum for 30 min, and then incubated with primary antibodies against TRAP (1:100, PA5-116970, Invitrogen), osteocalcin (1:100, M173, Takara), sclerostin (1:100, PA5-46977, Invitrogen) or Tau (1:100, 10274-1-AP, ProteinTech) overnight at 4 °C. Following this, sections were inbubated with the corresponding secondary antibodies for 1 h in the dark, followed by counterstaining with DAPI. Images were obtained using the Zeiss microscope, and the quantification was performed with ImageJ software.

### Collection of human bone samples

Human samples were obtained from patients underwent operations at Daping Hospital (Chongqing, China). In order to eliminate the effects of estrogen, all samples were collected from men patients. Osteoporosis was defined based on BMD measured by dual-energy X-ray absorptiometry (DXA, GE Health), according to standards (BMD T value <−2.5). Subjects with secondary osteoporosis, malignancy, hypertension, diabetes and other metabolic syndromes, cognition impairment were excluded from our study. Normal control human bone tissues were obtained from patients with fracture or suffered traumatic amputation (*n* = 5, aged from 35 to 40 years old), and bone tissues of osteoporosis patients were obtained from patients who underwent joint replacement (*n* = 6, aged from 60 to 80 years old). The study was performed in accordance with the Declaration of Helsinki and approved by the Ethics Committees of Daping Hospital (ID: #2019-127-01). Participants provided written informed consent to participate in the study.

### Statistical analyses

Statistical significance was determined using GraphPad Prism 9 (GraphPad). Statistical significance between two groups was determined using a two-tailed, unpaired Student’s *t*-test. For comparison of more than two groups, ANOVA analysis was used with Bonferroni post-hoc test as described in the figure legends. *P* values were considered significant if *P* < 0.05. All data points represent biological replicates, unless otherwise indicated in the figure legends. All data are presented as means ± SD unless otherwise stated in the figure legends. All specific statistical details can be found in the figure captions.

## Supplementary information

Supplementary information, Figure S1

Supplementary information, Fig. S2. Dexamethasone reduced osteoblast number independent of Tau in CIA and GIO models.

Supplementary information, Fig. S3. Tau Deficiency in female mice recapitulates its effect in male GIO model.

Supplementary information, Fig. S4. The effects of high-dose dexamethasone on apoptosis and osteoblastogenesis of bone marrow-derived mesenchymal stem cells in vitro.

Supplementary information, Fig. S5. FDA-approved drug library screen leads to the identification of TRx0237 as a drug that inhibits dexamethasone induced p-Tau S422 and osteoclastogenesis.

Supplementary information, Fig. S6. TRx0237 protects against GIO in a Tau-dependent manner.

Supplementary information, Fig. S7. TRx0237 is therapeutic against glucocorticoid-induced osteoporosis through inhibition of p-Tau Ser422.

Supplementary information, Fig. S8. TRx0237’s efficacy to protect against bone loss in GIO model.

Supplementary information, Table S1. The list of potential transcription factors that bind to Tau in response to high-dose dexamethasone
