# Supplementary material for: Tau is a receptor with low affinity for glucocorticoids and is required for glucocorticoid-induced bone loss
Source: Cell Res. 2025 Jan 2;35(1):23–44. doi: 10.1038/s41422-024-01016-0 (PMC11701132; doi:10.1038/s41422-024-01016-0)
Supplement: Supplementary file 1 — Supplementary information, Figure S1 [file 41422_2024_1016_MOESM1_ESM.pdf]

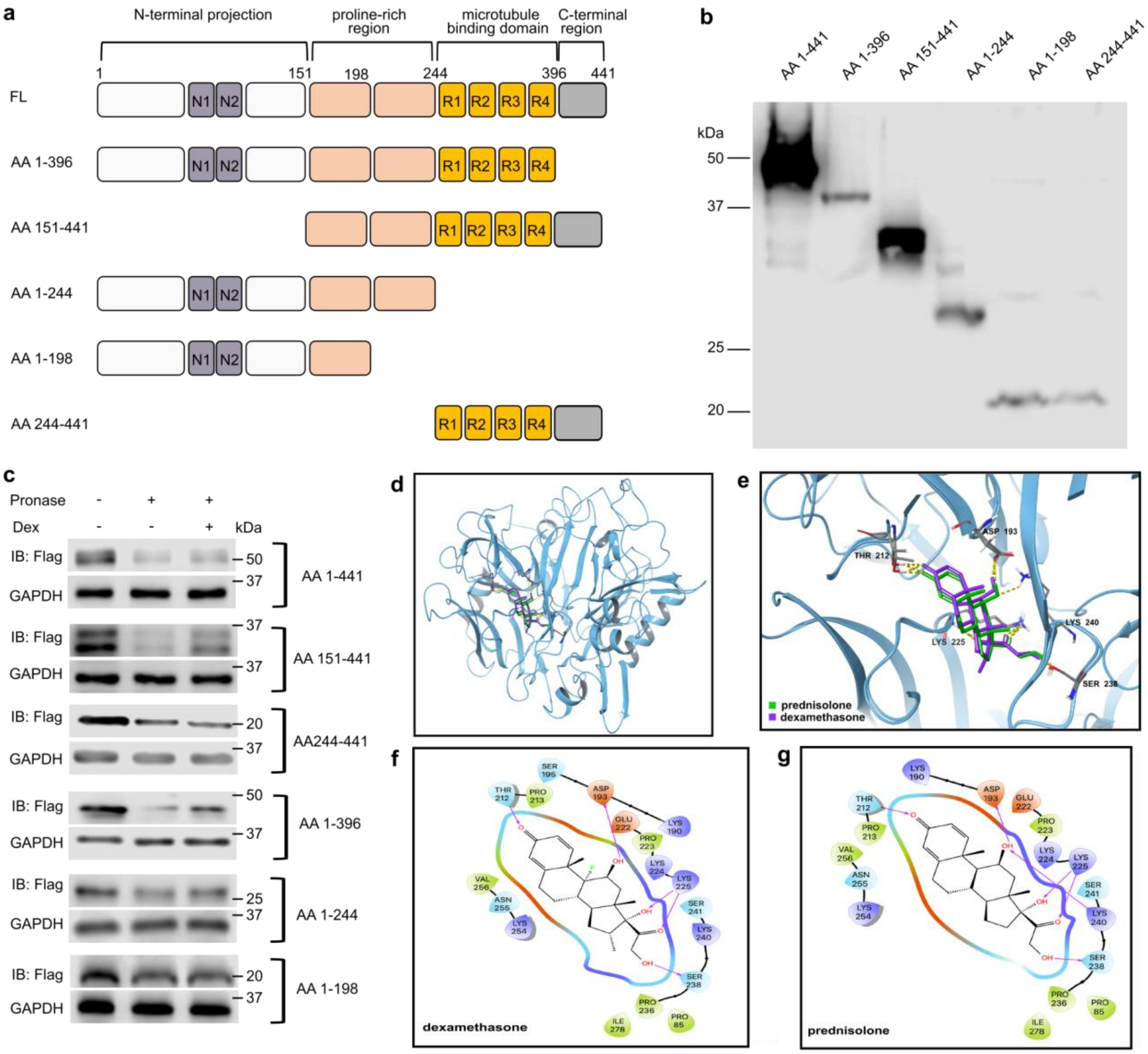

h

Ligand	Docking score (kcal/mol)	Amino acid residue interactions	Type of interaction	Distance (Å)
**dexamethasone**	-7.168	Asp193	Hydrogen bond	1.89
		Thr212	Hydrogen bond	1.94
		Lys225	Hydrogen bond	2.44 (CO...Lys225); 2.65 (HO...Lys225)
		Ser238	Hydrogen bond	2.25
**prednisolone**	-7.439	Asp193	Hydrogen bond	2.18
		Thr212	Hydrogen bond	1.92
		Lys225	Hydrogen bond	2.17 (CO...Lys225) 2.76 (HO...Lys225)
		Ser238	Hydrogen bond	1.97
		Ser240	Hydrogen bond	2.42

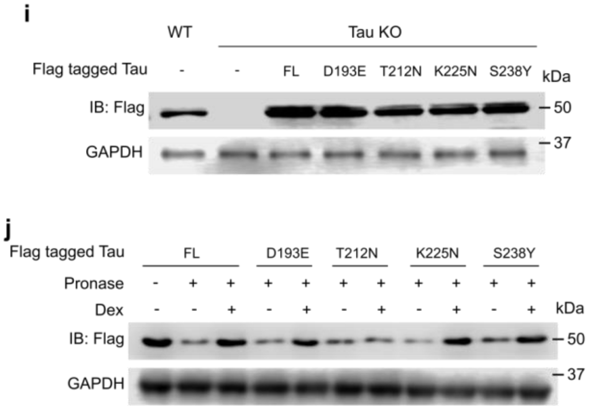

Supplementary information, Fig. S1. Threonine at position 212 (Thr212) in Tau is critical for dexamethasone binding. a Scheme of constructs encoding full length (FL) and serial deletion mutants of Tau. **b** Expressions of Flag-tagged full length (FL) and deletion mutants of Tau. HEK293T cells transfected with Flag-tagged FL and deletion mutants of Tau are lyzed and immunoblotted with anti-Flag antibody. **c** DARTS assay with FL and mutants of Tau. Cell lysates of FL and deletion mutants of Tau transfected HEK293T cells are incubated with 10μM dexamethasone and subjected to proteinase digestion, followed by immunoblotting with anti-Flag antibody. **d, e** Overview of the binding position of dexamethasone (violet) and prednisolone (green) in Tau protein. The Tau protein is depicted as cyan ribbons, and the important interacting residues are shown as sticks. Hydrogen bonds are represented by dotted yellow lines. **f, g** The 2D ligand-receptor interaction diagram of dexamethasone (**f**) and prednisolone (**g**) with important interactions shown. The amino acids within 4 Å are shown as colored bubbles, where green indicates hydrophobic residues, cyan indicates polar residues, red indicates negatively charged residues, and violet indicates positively charged residues. The magenta arrows show the hydrogen bonds. **h** Docking score and interacting residues in induced-fit docking predicted docking complexes of Tau with dexamethasone and prednisolone. **i** Expressions of Tau in WT and Tau knockout Raw 264.7 cells transfected with FL or indicated Tau point mutants, determined by immunoblotting with anti-Tau antibody. **j** DARTS assay with serial point mutations of Tau. Lysates of Tau knockout Raw264.7 cells transfected with FL or various Tau point mutants, as indicated, are incubated with 10μM dexamethasone and subjected to proteinase digestion, followed by immunoblotting with anti-Flag antibody. Representative image of duplicate results is shown.
