# Supplementary material for: Tau is a receptor with low affinity for glucocorticoids and is required for glucocorticoid-induced bone loss
Source: Cell Res. 2025 Jan 2;35(1):23–44. doi: 10.1038/s41422-024-01016-0 (PMC11701132; doi:10.1038/s41422-024-01016-0)
Supplement: Supplementary file 2 — Supplementary information, Fig. S2. Dexamethasone reduced osteoblast number independent of Tau in CIA and GIO models. [file 41422_2024_1016_MOESM2_ESM.pdf]

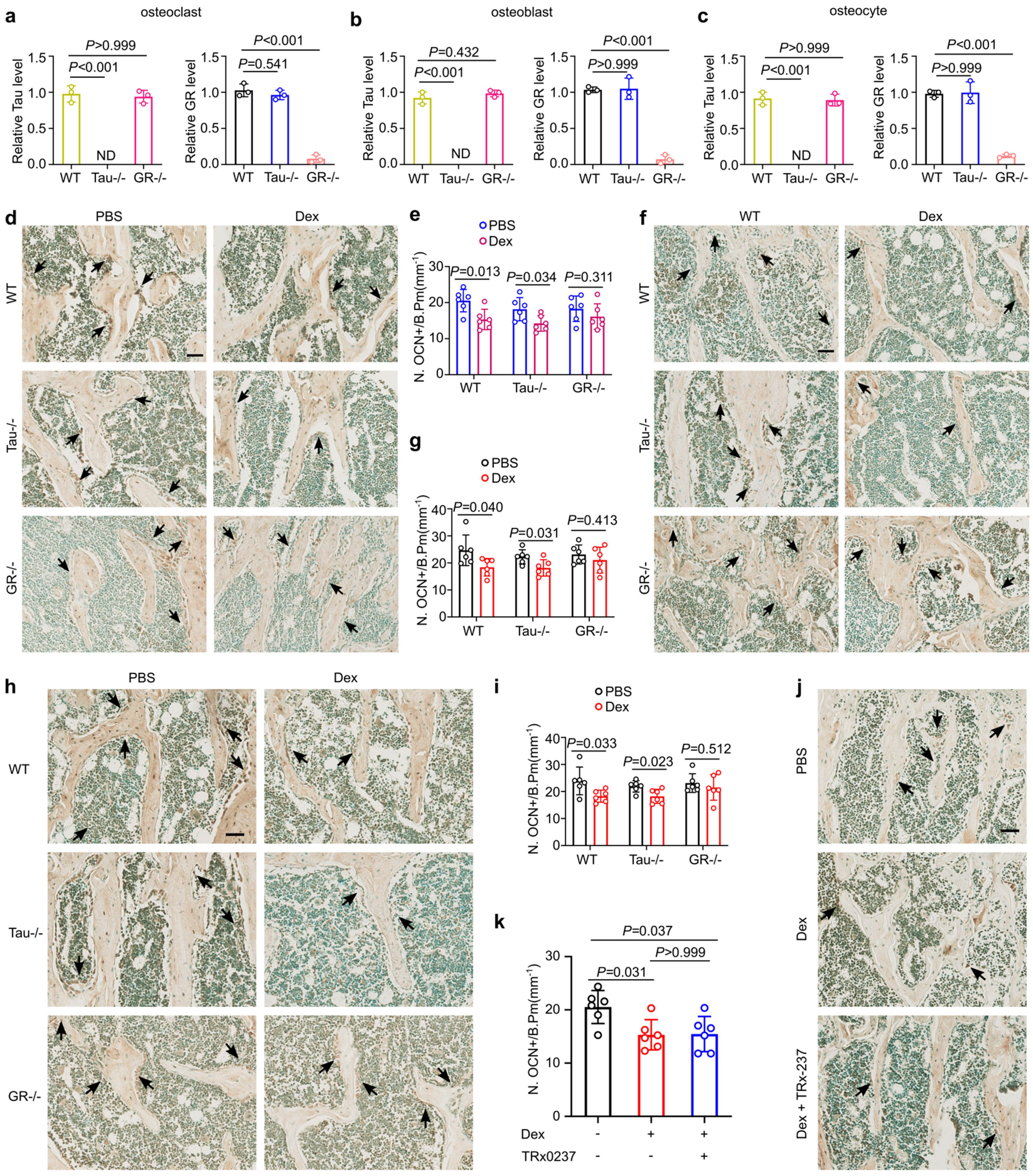

Supplementary information, Fig. S2. Dexamethasone reduced osteoblast number independent of Tau in CIA and GIO models. a-c Relative mRNA levels of *Tau* and *GR* in osteoclasts (a), osteoblasts (b) and osteocytes (c) isolated from WT, Tau-/- and GR-/- mice, assayed by qRT-PCR (n = 3 biological replicates, and

cells from three individual mouse were pooled together for each biological replicates). **d, e** Immunohistochemical staining of osteocalcin (OCN, stained brown) (d) and number of OCN positive cells per bone perimeter (N.Ob/B.Pm) (e) on femur trabecular bone of WT, Tau-/- and GR-/- mice with CIA treated with or without dexamethasone for 5 weeks (n = 6 mice for each group). **f, g** Immunohistochemical staining of OCN (f) and N.Ob/B.Pm (g) on femur trabecular bone of WT, Tau-/- and GR-/- male mice treated with or without dexamethasone for 5 weeks (n = 6 mice for each group). **h, i** Immunohistochemical staining of OCN (h) and N.Ob/B.Pm (i) on femur trabecular bone of WT, Tau-/- and GR-/- female mice treated with or without dexamethasone for 5 weeks (n = 6 mice for each group). **j, k** Immunohistochemical staining of OCN (j) and N.Ob/B.Pm (k) on femur trabecular bone of WT male CIA mice with indicated treatment (n = 6 mice for each group) . Scale bar = 50 μm. Data are mean ± SD, P values are calculated by two-tailed unpaired Student's t-test (e, g, and i) and by one way ANOVA with Bonferroni post-hoc test (a-c and k).
