# Supplementary material for: Tau is a receptor with low affinity for glucocorticoids and is required for glucocorticoid-induced bone loss
Source: Cell Res. 2025 Jan 2;35(1):23–44. doi: 10.1038/s41422-024-01016-0 (PMC11701132; doi:10.1038/s41422-024-01016-0)
Supplement: Supplementary file 3 — Supplementary information, Fig. S3. Tau Deficiency in female mice recapitulates its effect in male GIO model. [file 41422_2024_1016_MOESM3_ESM.pdf]

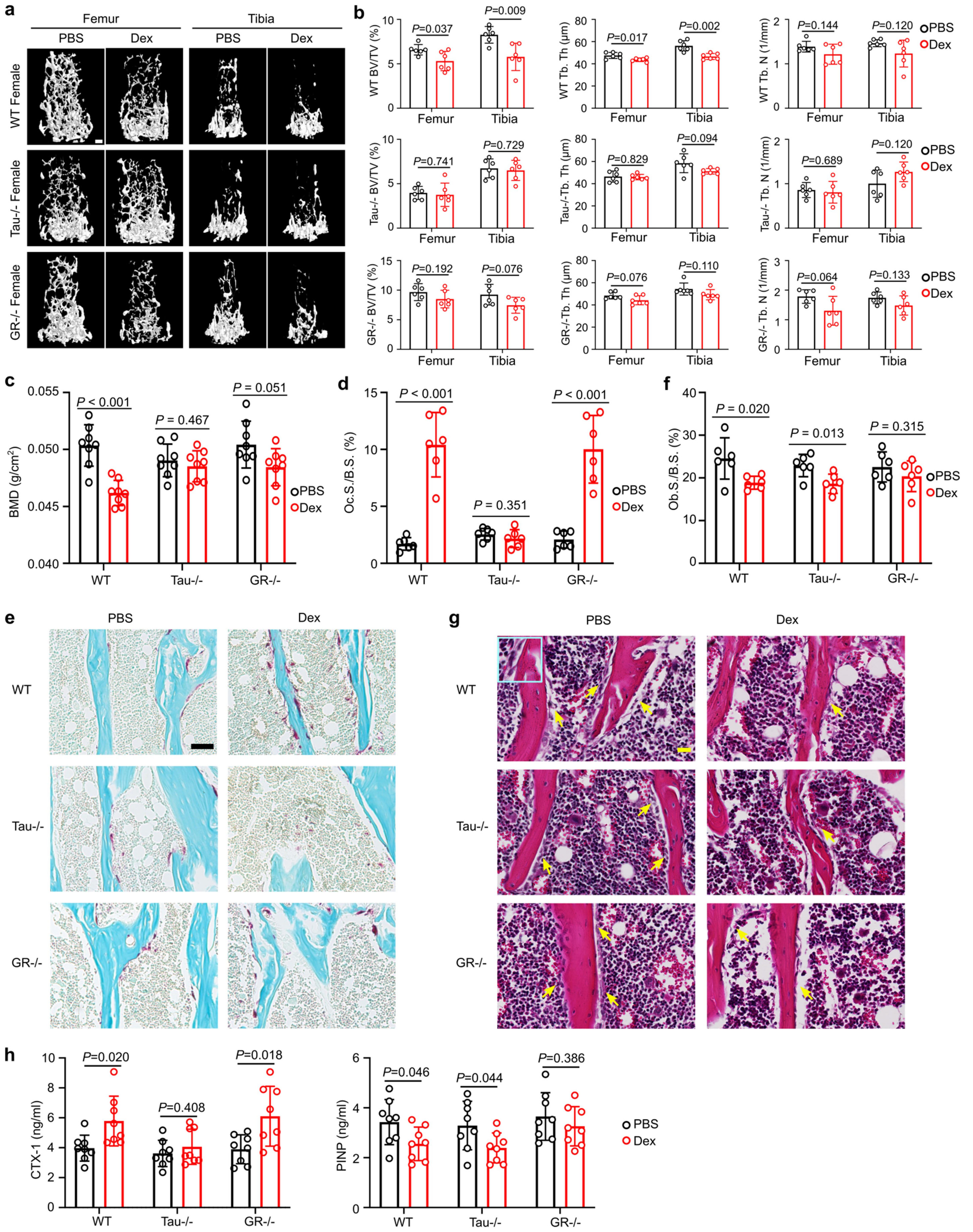

Supplementary information, Fig. S3. Tau Deficiency in female mice recapitulates its effect in male GIO

model. a Representative reconstructed 3D micro-CT images of femur and tibia trabecular bone of WT, Tau-/- and GR-/- female mice with or without GIO. Scale bar = 250 µm. **b** Quantification of trabecular parameters including BV/TV, Tb. Th and Tb. N in WT, Tau-/- and GR-/- female mice with or without GIO (n = 8 mice for each group). **c** Whole body bone mineral density in WT, Tau-/- and GR-/- female mice treated with or without dexamethasone for 5 weeks, measured by DEXA scanning (n = 8 mice for each group). **d, e** Quantification of TRAP+ osteoclast surface per bone surface (Oc.S./B.S.) (d) and representative TRAP staining image (**e**), of the femur distal metaphysis of WT, Tau-/- and GR-/- female mice in the same experiment (n = 6 mice for each group). Scale bar = 20 µm. **f** Quantification of osteoblast surface per bone surface (Ob.S./B.S.) in the indicated mice. **g** Representative H&E stained images of femur showing osteoblasts (yellow arrows) on the trabecular bone. The area defined by blue rectangle on representative image is enlarged as inset. Scale bar = 50 µm. **h** Serum levels of CTX-1 and PINP in the indicated female mice, assayed with ELISA (n = 8 mice for each group). Data are mean ± SD, *P* values are calculated by two-tailed unpaired Student's *t*-test.
