# Supplementary material for: Tau is a receptor with low affinity for glucocorticoids and is required for glucocorticoid-induced bone loss
Source: Cell Res. 2025 Jan 2;35(1):23–44. doi: 10.1038/s41422-024-01016-0 (PMC11701132; doi:10.1038/s41422-024-01016-0)
Supplement: Supplementary file 4 — Supplementary information, Fig. S4. The effects of high-dose dexamethasone on apoptosis and osteoblastogenesis of bone marrow-derived mesenchymal stem cells in vitro. [file 41422_2024_1016_MOESM4_ESM.pdf]

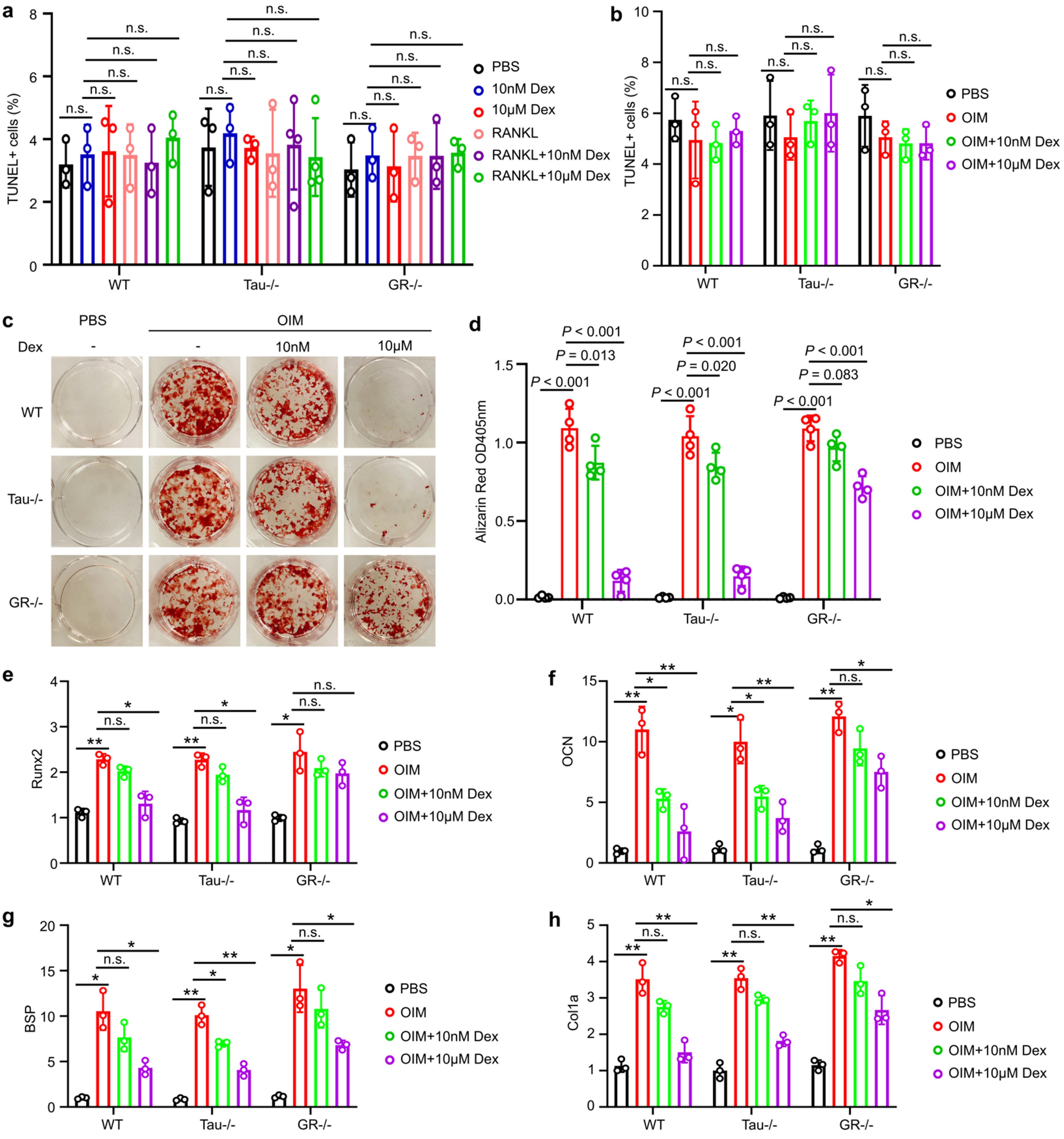

a
TUNEL+ cells (%)
n.s.
WT
Tau-/-
GR-/-
PBS
10nM Dex
10μM Dex
RANKL
RANKL+10nM Dex
RANKL+10μM Dex
b
TUNEL+ cells (%)
PBS
OIM
OIM+10nM Dex
OIM+10μM Dex
c
PBS
OIM
Dex
-
10nM
10μM
WT
Tau-/-
GR-/-
d
Alizarin Red OD405nm
P < 0.001
P = 0.013
P = 0.020
P = 0.083
e
Runx2
f
OCN
g
BSP
h
Col1a

Supplementary information, Fig. S4. The effects of high dose dexamethasone on apoptosis and osteoblastogenesis of bone marrow-derived mesenchymal stem cells *in vitro*. a Apoptosis in bone marrow derived macrophage isolated from WT, Tau-/- and GR -/- mice, cultured with 20 ng/ml M-CSF along with or without 50 ng/ml RANKL and/or different dose of dexamethasone for 5 days, evaluated by TUNEL assay (n = 3). **b** Apoptosis in bone marrow cells isolated from WT, Tau-/- and GR-/- mice, stimulated without or with osteo-induction media (OIM) in the presence of different dose of dexamethasone for 5 days, evaluated by TUNEL assay (n = 3). **c, d** *In vitro* osteoblastogenesis of primary WT, Tau-/- and GR-/- bone marrow cells after osteogenic induction with OIM and different dose of dexamethasone for 21 days, determined with Alizarin Red staining. Representative sating (**c**) and quantification of osteoblastogenesis (**d**) (n = 4). **e-h** The expressions of osteogenesis-related genes *Runx2* (e) (1 day of differentiation), and *OCN* (f), *BSP* (**g**) and *Col1a* (**h**) (5 days of differentiation), determined with qRT-PCR (n = 3). Data are mean ± SD, *P* values are calculated by one way ANOVA with Bonferroni post-hoc test.
