# Supplementary material for: Tau is a receptor with low affinity for glucocorticoids and is required for glucocorticoid-induced bone loss
Source: Cell Res. 2025 Jan 2;35(1):23–44. doi: 10.1038/s41422-024-01016-0 (PMC11701132; doi:10.1038/s41422-024-01016-0)
Supplement: Supplementary file 5 — Supplementary information, Fig. S5. FDA-approved drug library screen leads to the identification of TRx0237 as a drug that inhibits dexamethasone induced p-Tau S422 and osteoclastogenesis. [file 41422_2024_1016_MOESM5_ESM.pdf]

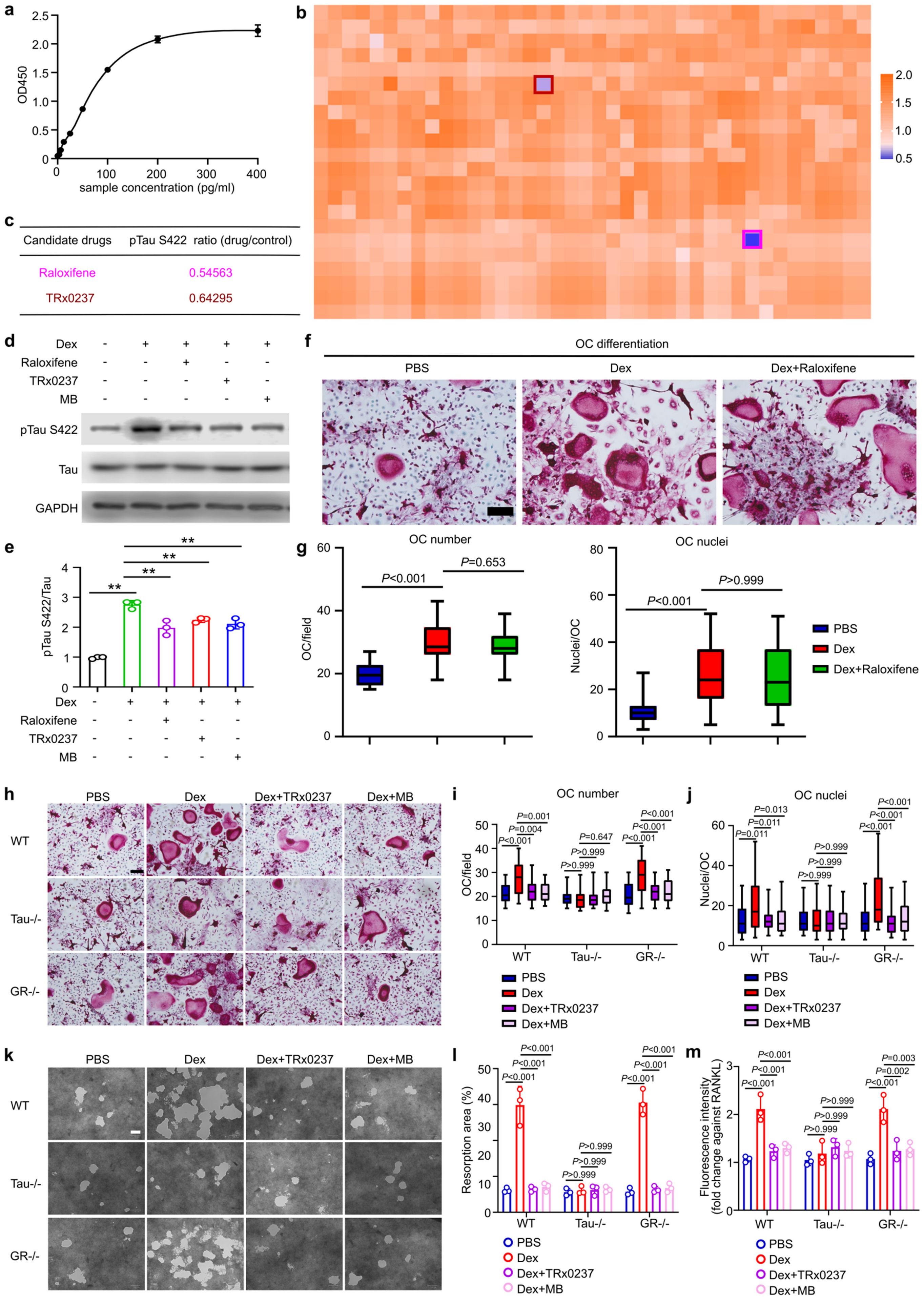

a
OD450
sample concentration (pg/ml)
b
c
Candidate drugs
pTau S422 ratio (drug/control)
Raloxifene 0.54563
TRx0237 0.64295
d
Dex
Raloxifene
TRx0237
MB
pTau S422
Tau
GAPDH
e
pTau S422/Tau
f
OC differentiation
PBS
Dex
Dex+Raloxifene
g
OC number
OC nuclei
OC/field
Nuclei/OC
P<0.001
P=0.653
P>0.999
h
Dex+TRx0237
Dex+MB
WT
Tau-/-
GR-/-
i
j
k
l
Resorption area (%)
m
Fluorescence intensity (fold change against RANKL)

Supplementary information, Fig. S5. FDA-approved drug library screen leads to the identification of TRx0237 as a drug that inhibits dexamethasone induced p-Tau S422 and osteoclastogenesis. a Development of ELISA-based high-throughput screening assay. Immobilized 12.5 ng Tau antibody binds to a serial dilution of biotinylated anti-phosphorylated Tau Ser422 antibody (0 - 400 pg/ml) (n = 3). **b** Heatmap illustration of effects of drugs on 10 μM dexamethasone stimulation of Tau phosphorylation at Ser422, represented by normalized OD450 fold change relative to no drug control. The colors indicate drug activity, and color intensity represents potency. **c** Identities of two candidate drugs that show inhibition of dexamethasone induced p-Tau S422 from ELISA based screen. **d, e** Immunoblotting analysis (**d**) and quantification (**e**) of p-Tau S422 in 10μM dexamethasone stimulated 1 μM drug pretreated THP-1 cells (n = 3). **f** Representative bright-field images of TRAP-positive bone marrow macrophage derived osteoclasts *in vitro*. Primary WT bone marrow derived macrophages are differentiated with 20 ng/ml M-CSF and 50 ng/ml RANKL supplemented with 10 μM dexamethasone and 1 μM indicated drug for 7 days. Scale bar = 100 μm. **g** Number of TRAP+ multinuclear osteoclasts (n = 20, number per field of view, from three mice per group) and nuclei per osteoclast a (n = 25 per group) shown in **f**. **h** Representative microscopic images of TRAP staining of bone marrow macrophage derived osteoclasts. Bone marrow derived macrophages isolated from WT, Tau-/- and GR-/- mice are differentiated with 20 ng/ml M-CSF and 50 ng/ml RANKL supplemented with high dose of dexamethasone and 1 μM TRx0237 or methylene blue (MB) for 7 days. Scale bar = 100 μm. **i, j** Quantification of number of TRAP+ multinuclear osteoclast (n = 20, number per field of view, from three mice per age group) **(i)** and nuclei per osteoclast (n = 25 per group) **(i)**. **k** Representative microscopic images of resorption activity of osteoclast grown on OsteoAssay plates *in vitro* in the same experiment. **(l)** Quantification of the resorption pit areas shown in **k**. **m** Quantification of fluorescence intensity released into media in osteoclasts derived from WT, Tau-/- and GR-/- mouse bone marrow macrophages cultured with 20 ng/ml M-CSF and 50 ng/ml RANKL supplemented with 10 μM dexamethasone and 1 μM TRx0237 or methylene blue (MB) (n = 3) for 7 days. Data are mean ± SD, *P* values are calculated by one way ANOVA with Bonferroni post-hoc test.
