# Supplementary material for: Tau is a receptor with low affinity for glucocorticoids and is required for glucocorticoid-induced bone loss
Source: Cell Res. 2025 Jan 2;35(1):23–44. doi: 10.1038/s41422-024-01016-0 (PMC11701132; doi:10.1038/s41422-024-01016-0)
Supplement: Supplementary file 7 — Supplementary information, Fig. S7. TRx0237 is therapeutic against glucocorticoid-induced osteoporosis through inhibition of p-Tau Ser422. [file 41422_2024_1016_MOESM7_ESM.pdf]

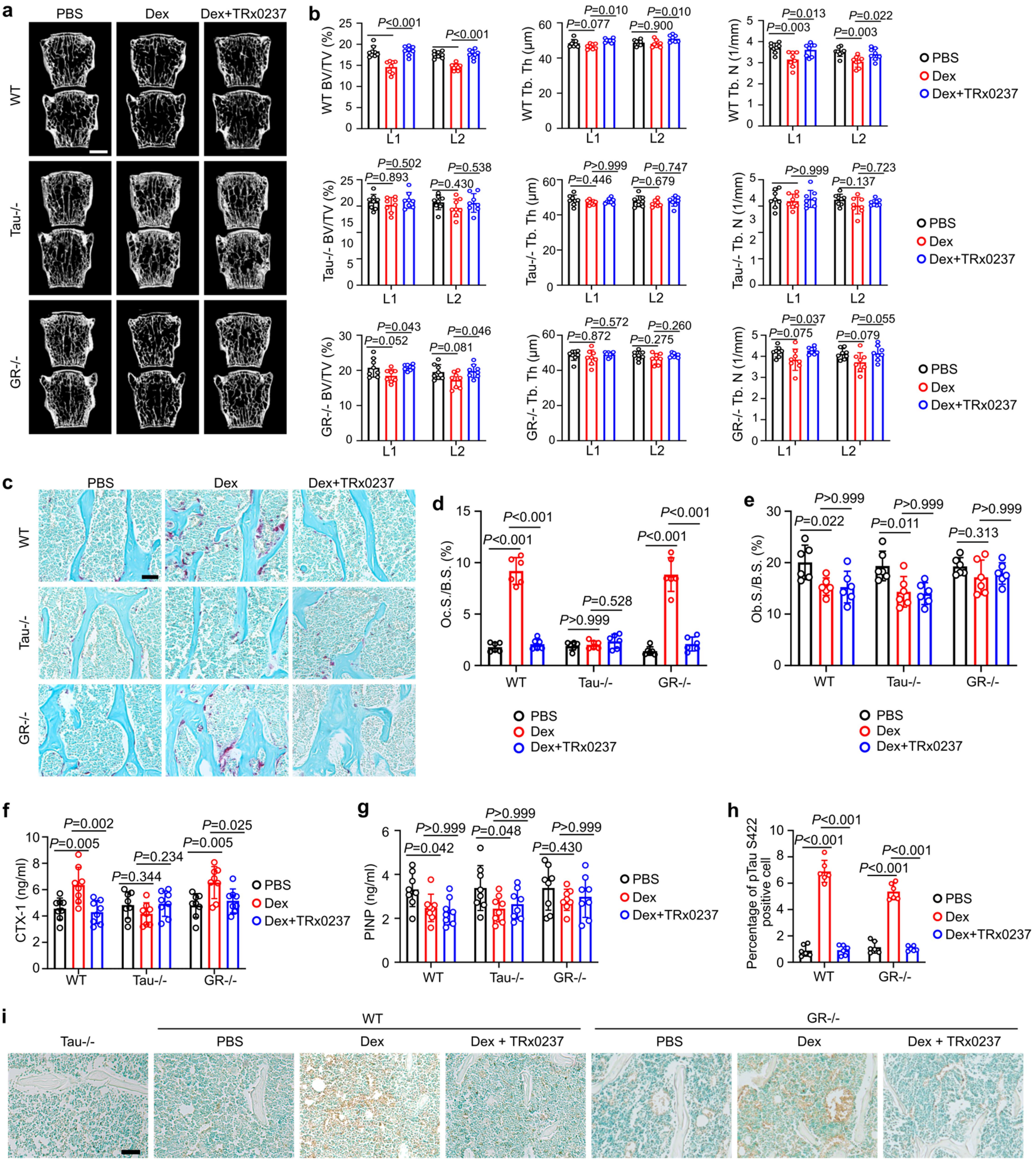

a
PBS
Dex
Dex+TRx0237
WT
Tau-/-
GR-/-
b
WT BV/TV (%)
WT Tb. Th (μm)
WT Tb. N (1/mm)
Tau-/- BV/TV (%)
Tau-/- Tb. Th (μm)
Tau-/- Tb. N (1/mm)
GR-/- BV/TV (%)
GR-/- Tb. Th (μm)
GR-/- Tb. N (1/mm)
L1
L2
P<0.001
P=0.010
P=0.077
P=0.900
P=0.013
P=0.003
P=0.022
P=0.502
P=0.893
P=0.538
P=0.430
P>0.999
P=0.446
P=0.747
P=0.679
P>0.999
P=0.723
P=0.137
P=0.043
P=0.052
P=0.046
P=0.081
P=0.572
P=0.872
P=0.260
P=0.275
P=0.037
P=0.075
P=0.055
P=0.079
PBS
Dex
Dex+TRx0237
c
d
Oc.S./B.S. (%)
P<0.001
P>0.999
P=0.528
e
Ob.S./B.S. (%)
P>0.999
P=0.022
P=0.011
P=0.313
f
CTX-1 (ng/ml)
P=0.002
P=0.005
P=0.234
P=0.344
P=0.025
g
PINP (ng/ml)
P>0.999
P=0.042
P=0.048
P=0.430
h
Percentage of pTau S422 positive cell
P<0.001
i
Tau-/-
WT
GR-/-
Dex + TRx0237

Supplementary information, Fig. S7. TRx0237 is therapeutic against glucocorticoid-induced osteoporosis through inhibition of p-Tau Ser422. a, b Representative reconstructed 3D micro-CT images (**a**) and quantification (**b**) of trabecular bone of the L1 and L2 vertebra from WT, Tau-/- and GR-/- male mice treated with or without dexamethasone in the presence or absence of TRx0237, as indicated, for 5 weeks (n = 8 mice for each group). Scale bar = 1 mm in **a**. **c, d** Representative TRAP staining image (**c**) and corresponding quantification of TRAP+ Oc.S./B.S. (**d**) of the lumbar vertebrae of indicated mice (n = 6 mice for each group). Sale bar = 50 µm in c. **e** Quantification of osteoblast surface per bone surface (Ob.S./B.S.) in the lumbar vertebrae of the indicated mice. **f, g** CTX-1 level (**f**), and PINP level (**g**) in sera isolated from WT, Tau-/- and GR-/- male mice treated with or without 10 mg/kg body weight dexamethasone and 4 mg/kg body weight TRx0237 for 5 weeks, assayed by ELISA (n = 8 mice for each group). **h** Quantification of pTauSer422 positive cells in femur of indicated mice in the same experiment. **i** Representative immunohistochemistry staining of pTauSer422 in femur of indicated mice (n = 6 mice for each group). Scale bar = 50 µm. Data are mean ± SD, *P* values are calculated by one way ANOVA with Bonferroni post-hoc test.
