# Supplementary material for: Tau is a receptor with low affinity for glucocorticoids and is required for glucocorticoid-induced bone loss
Source: Cell Res. 2025 Jan 2;35(1):23–44. doi: 10.1038/s41422-024-01016-0 (PMC11701132; doi:10.1038/s41422-024-01016-0)
Supplement: Supplementary file 8 — Supplementary information, Fig. S8. TRx0237’s efficacy to protect against bone loss in GIO model. [file 41422_2024_1016_MOESM8_ESM.pdf]

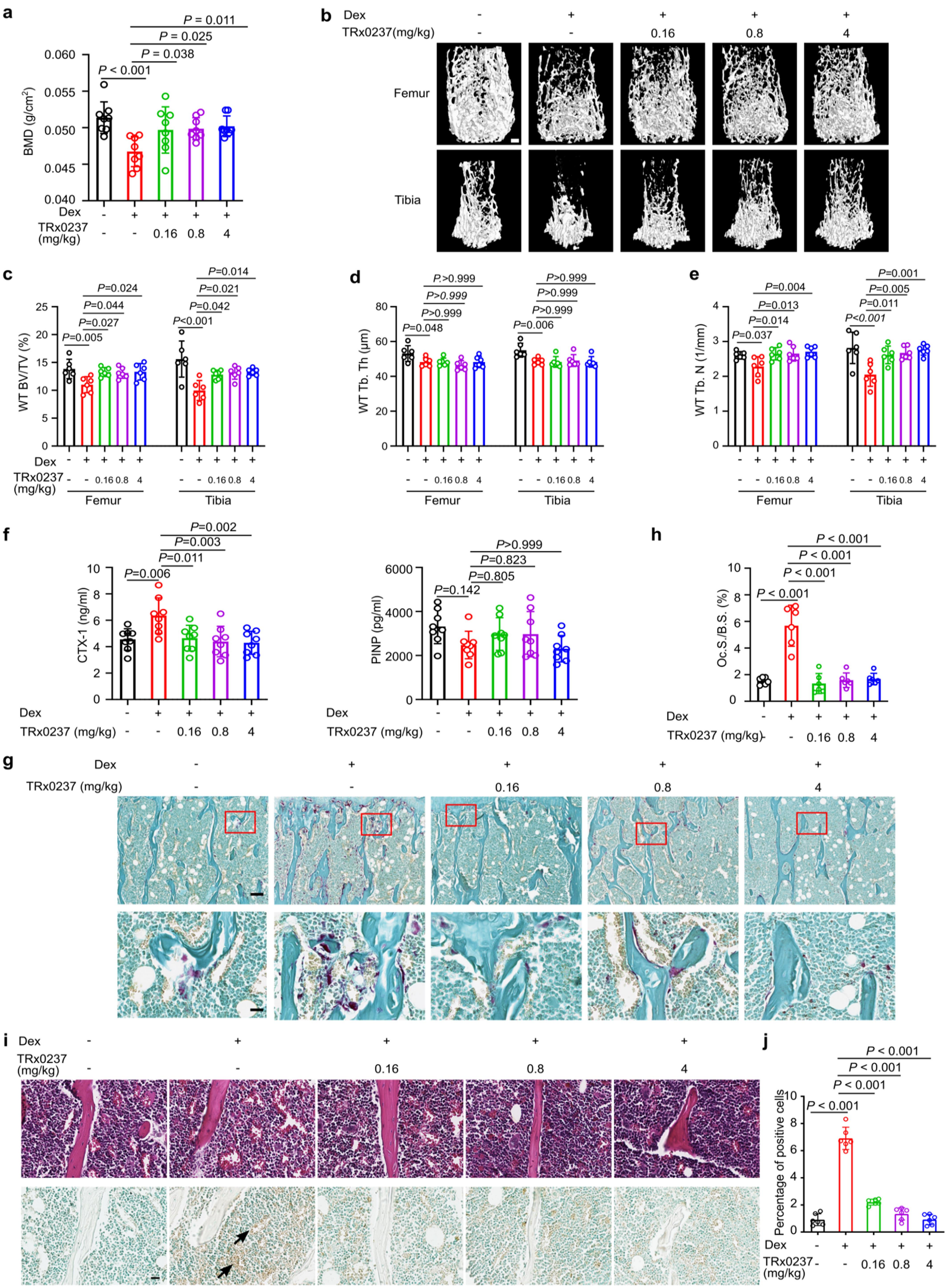
a
P = 0.011
P = 0.025
P = 0.038
P < 0.001
BMD (g/cm²)
0.060
0.055
0.050
0.045
0.040
Dex - + + + +
TRx0237 (mg/kg) - - 0.16 0.8 4
b
Dex - + + + +
TRx0237(mg/kg) - - 0.16 0.8 4
Femur
Tibia
c
P=0.024
P=0.044
P=0.027
P=0.005
P=0.014
P=0.021
P=0.042
P<0.001
WT BV/TV (%)
Dex
TRx0237 (mg/kg)
0.16 0.8 4
Femur
Tibia
d
P.>0.999
P>0.999
P>0.999
P=0.048
P>0.999
P>0.999
P>0.999
P=0.006
WT Tb. Th (μm)
Femur
Tibia
e
P=0.004
P=0.013
P=0.014
P=0.037
P=0.001
P=0.005
P=0.011
P<0.001
WT Tb. N (1/mm)
Femur
Tibia
f
P=0.002
P=0.003
P=0.011
P=0.006
CTX-1 (ng/ml)
Dex - + + + +
TRx0237 (mg/kg) - - 0.16 0.8 4
P>0.999
P=0.823
P=0.805
P=0.142
PINP (pg/ml)
Dex - + + + +
TRx0237 (mg/kg) - - 0.16 0.8 4
h
P < 0.001
P < 0.001
P < 0.001
P < 0.001
Oc.S./B.S. (%)
Dex - + + + +
TRx0237 (mg/kg) - - 0.16 0.8 4
g
Dex - + + + +
TRx0237 (mg/kg) - - 0.16 0.8 4
i
Dex - + + + +
TRx0237 (mg/kg) - - 0.16 0.8 4
j
P < 0.001
P < 0.001
P < 0.001
P < 0.001
Percentage of positive cells
Dex - + + + +
TRx0237 (mg/kg) - - 0.16 0.8 4

Supplementary information, Fig. S8. TRx0237's efficacy to protect against bone loss in GIO model. a Whole body bone mineral density in WT male mice treated with or without dexamethasone and serial doses of TRx0237 for 5 weeks, measured by DEXA scanning (n = 8 mice for each group). **b** Representative reconstructed 3D micro-CT images of trabecular bone of femur and tibia of WT male mice treated with dexamethasone and difference dosage of TRx0237 (n = 6 mice for each group). **c-e** Quantification of trabecular, BV/TV (c), Tb. Th (d) and Tb. N (e) in WT male mice treated without or with dexamethasone along with different dose of TRx0237 for 5 weeks (n = 6 mice for each group). **f** Serum levels of CTX-1 and PINP in the indicated male mice, assayed by ELISA (n = 8 mice for each group). **g, h** Representative TRAP staining image (g) and quantification of TRAP+ Oc.S./B.S. (h) of distal metaphysis of the femur of WT male mice in the same experiment (n = 6 mice for each group). Images in lower panel (scale bar = 20 μm) are high-resolution versions of the boxed regions in upper panel images (scale bar = 100 μm). **i, j** H&E and immunohistochemistry staining (i), and corresponding quantification (j) of pTau S422 in femur of WT male mice in the same experiment (n = 6 mice for each group). Scale bar = 20 μm. Data are mean ± SD, *P* values are calculated by one way ANOVA with Bonferroni post-hoc test.
