# Supplementary material for: Tau is a receptor with low affinity for glucocorticoids and is required for glucocorticoid-induced bone loss
Source: Cell Res. 2025 Jan 2;35(1):23–44. doi: 10.1038/s41422-024-01016-0 (PMC11701132; doi:10.1038/s41422-024-01016-0)
Supplement: Supplementary file 9 — Supplementary information, Table S1. The list of potential transcription factors that bind to Tau in response to high-dose dexamethasone [file 41422_2024_1016_MOESM9_ESM.pdf]

Supplementary information, Table S1. The list of potential transcription factors that bind to Tau in response to high dose dexamethasone

Access #	Description
P25799-3	Nuclear factor NF-kappa-B p105 subunit
Q61164	Transcriptional repressor CTCF
Q8CGF7-2	Isoform 2 of Transcription elongation regulator 1
O55201-2	Isoform 2 of Transcription elongation factor SPT5
Q9D0D5	General transcription factor IIE subunit 1
